# Supplementary material for: uORF-targeting steric block antisense oligonucleotides do not reproducibly increase RNASEH1 expression
Source: Mol Ther Nucleic Acids. 2024 Nov 28;36(1):102406. doi: 10.1016/j.omtn.2024.102406 (PMC11697566; doi:10.1016/j.omtn.2024.102406)
Supplement: Document S1. Figures S1–S7, Tables S1, and S2 [file mmc1.pdf]

OMTN, Volume *36*

Supplemental information

uORF-targeting steric block antisense oligonucleotides do not reproducibly increase RNASEH1 expression

Nina Ahlskog, Nenad Svrzikapa, Rushdie Abuhamdah, Mahnseok Kye, Yahya Jad, Ning Feng, Britt Hanson, Matthew J.A. Wood, and Thomas C. Roberts

Supplemental Information

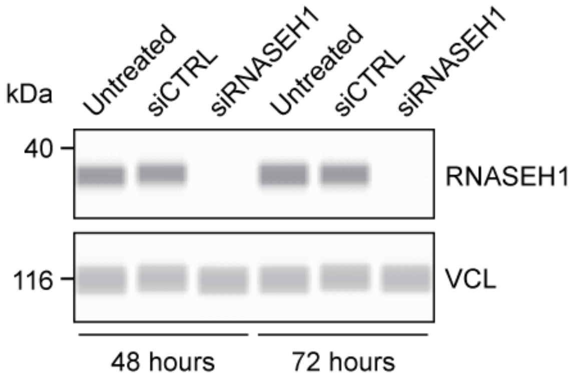

Figure S1

Anti-RNASEH1 antibody validation.

HeLa cells were transfected with a pool of siRNAs targeting RNASEH1, or a control siRNA pool and protein harvested 48 or 72 hours post transfection. RNASEH1 protein was quantified by Jess capillary western blot. Vinculin (VCL) was used as a loading control. RNASEH1 was detected at the expected size (32 kDa) and was undetectable after siRNA-mediated knockdown.

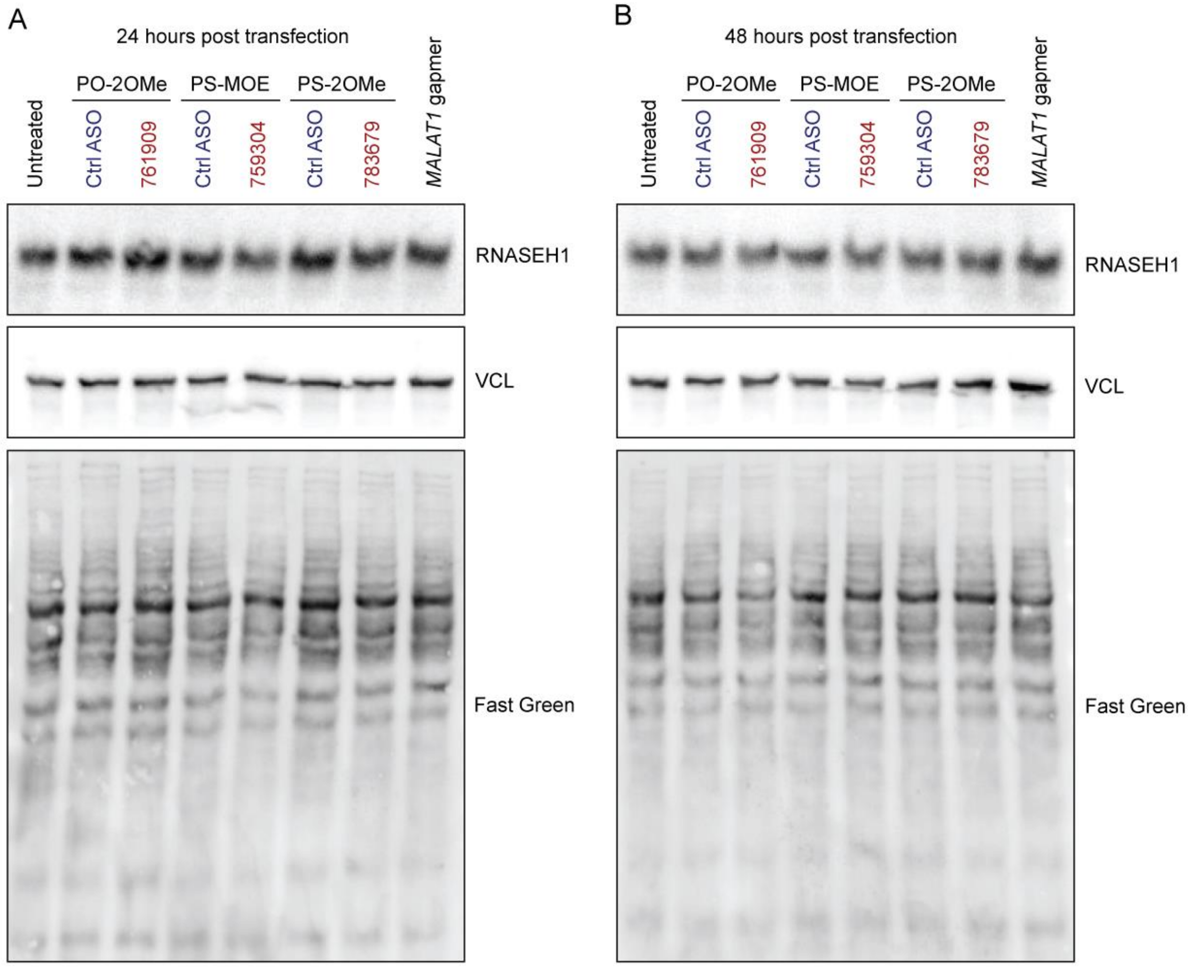

Figure S2

uORF-targeting steric block ASOs do not increase RNASEH1 protein expression at 24 and 48 hours post transfection as assessed by western blot.

HeLa cells were transfected with ASOs as indicated and protein harvested at (**A**) 24 hours, and (**B**) 48 hours post transfection. Samples were analysed by standard SDS-PAGE western blotting using anti-RNASEH1 antibodies. Vinculin (VCL) was used as a loading control protein, and total protein loading was assessed by Fast Green staining.

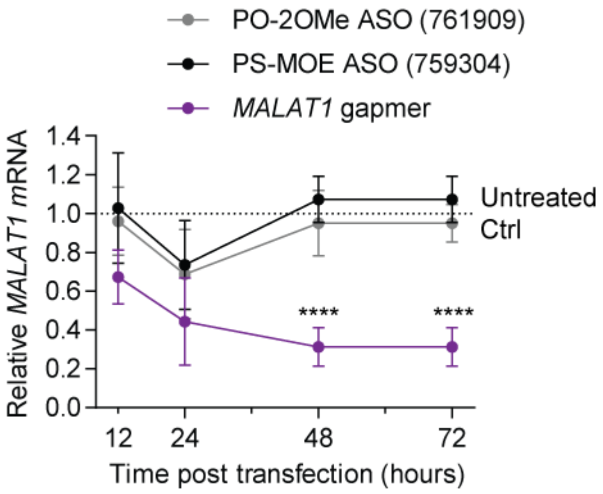

Figure S3

Validation of ASO transfection protocol.

HeLa cells were transfected with a gapmer (100 nM) ASO targeting *MALAT1* or non-MALAT1-targeting ASOs and RNA harvested at 12, 24, 48, and 72 hours post transfection. *MALAT1* transcript levels were determined by RT-qPCR and normalised to *RPL10* expression. Values are mean+SD. Untreated control samples were utilised as calibrator samples and were scaled to a value of 1 at each time point. Statistical differences were determined by one-way ANOVA and Tukey *post hoc* test performed at each time point. ***P<0.0001 (comparison of the *MALAT1* gapmer treatment versus the PS-MOE ASO), n=4 completely independent experiments.

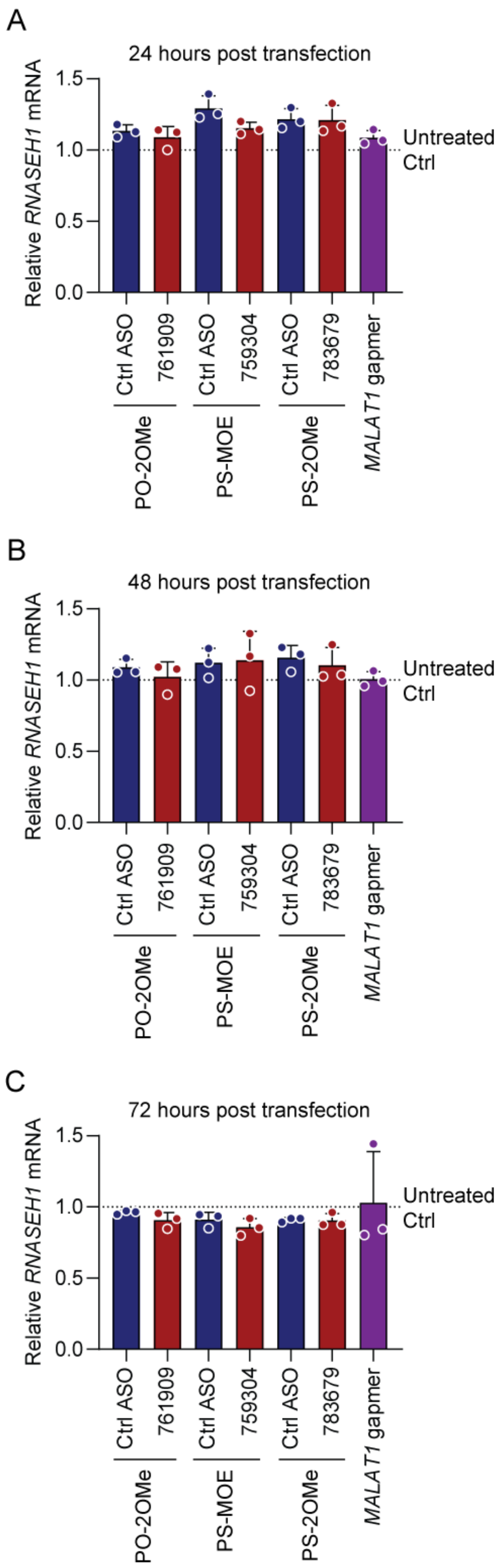
A
24 hours post transfection
Relative RNASEH1 mRNA
1.5
1.0
0.5
0.0
Untreated Ctrl
Ctrl ASO
761909
Ctrl ASO
759304
Ctrl ASO
783679
MALAT1 gapmer
PO-2OMe
PS-MOE
PS-2OMe
B
48 hours post transfection
Relative RNASEH1 mRNA
1.5
1.0
0.5
0.0
Untreated Ctrl
Ctrl ASO
761909
Ctrl ASO
759304
Ctrl ASO
783679
MALAT1 gapmer
PO-2OMe
PS-MOE
PS-2OMe
C
72 hours post transfection
Relative RNASEH1 mRNA
1.5
1.0
0.5
0.0
Untreated Ctrl
Ctrl ASO
761909
Ctrl ASO
759304
Ctrl ASO
783679
MALAT1 gapmer
PO-2OMe
PS-MOE
PS-2OMe

Figure S4

uORF-targeting steric block ASOs do not alter *RNASEH1* mRNA levels at 24, 48, and 72 hours post transfection.

HeLa cells were transfected with 100 nM ASOs or matched chemistry controls and cells harvested at (**A**) 24 hours, (**B**) 48 hours, or (**C**) 72 hours post transfection. *RNASEH1* transcript levels were determined by RT-qPCR and normalised to *RPL10* expression. The value of untreated control samples is indicated by the dotted line (scaled to a value of 1). A gapmer targeting *MALAT1* was included as a positive control for transfection, which is not expected to influence *RNASEH1* expression. Values are mean+SD. Statistical significance was assessed by paired Student's *t*-test between each treatment and its respective control ASO, (no significant changes detected), *n*=3 completely independent experiments.

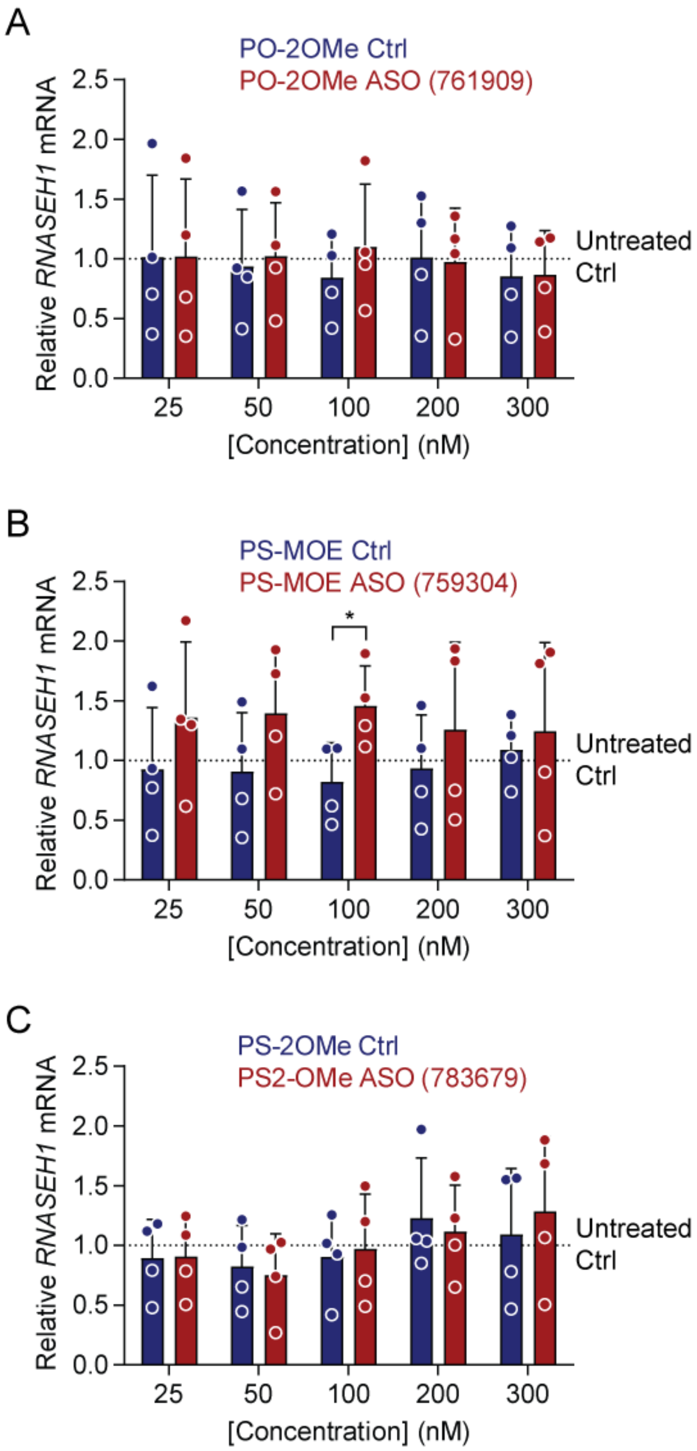
A
PO-2OMe Ctrl
PO-2OMe ASO (761909)
Relative RNASEH1 mRNA
2.5
2.0
1.5
1.0
0.5
0.0
25
50
100
200
300
[Concentration] (nM)
Untreated Ctrl
B
PS-MOE Ctrl
PS-MOE ASO (759304)
*
Relative RNASEH1 mRNA
[Concentration] (nM)
Untreated Ctrl
C
PS-2OMe Ctrl
PS2-OMe ASO (783679)
Relative RNASEH1 mRNA
[Concentration] (nM)
Untreated Ctrl

Figure S5

Expression of *RNASEH1* mRNA levels after treatment with uORF-targeting steric block ASOs at various doses.

HeLa cells were transfected with ASOs at concentrations as indicated and protein harvested after 48 hours for (**A**) PO-2OMe, (**B**) PS-MOE, and (**C**) PS-2OMe nucleic acid chemistries. *RNASEH1* transcript levels were determined by RT-qPCR and normalised to *RPL10* expression. The value of untreated control samples is indicated by the dotted line (scaled to a value of 1). Values are mean+SD. Statistical significance for protein data were assessed by paired Student's *t*-test within each oligonucleotide dose. *$P<0.05$, $n=4$ independent experiments

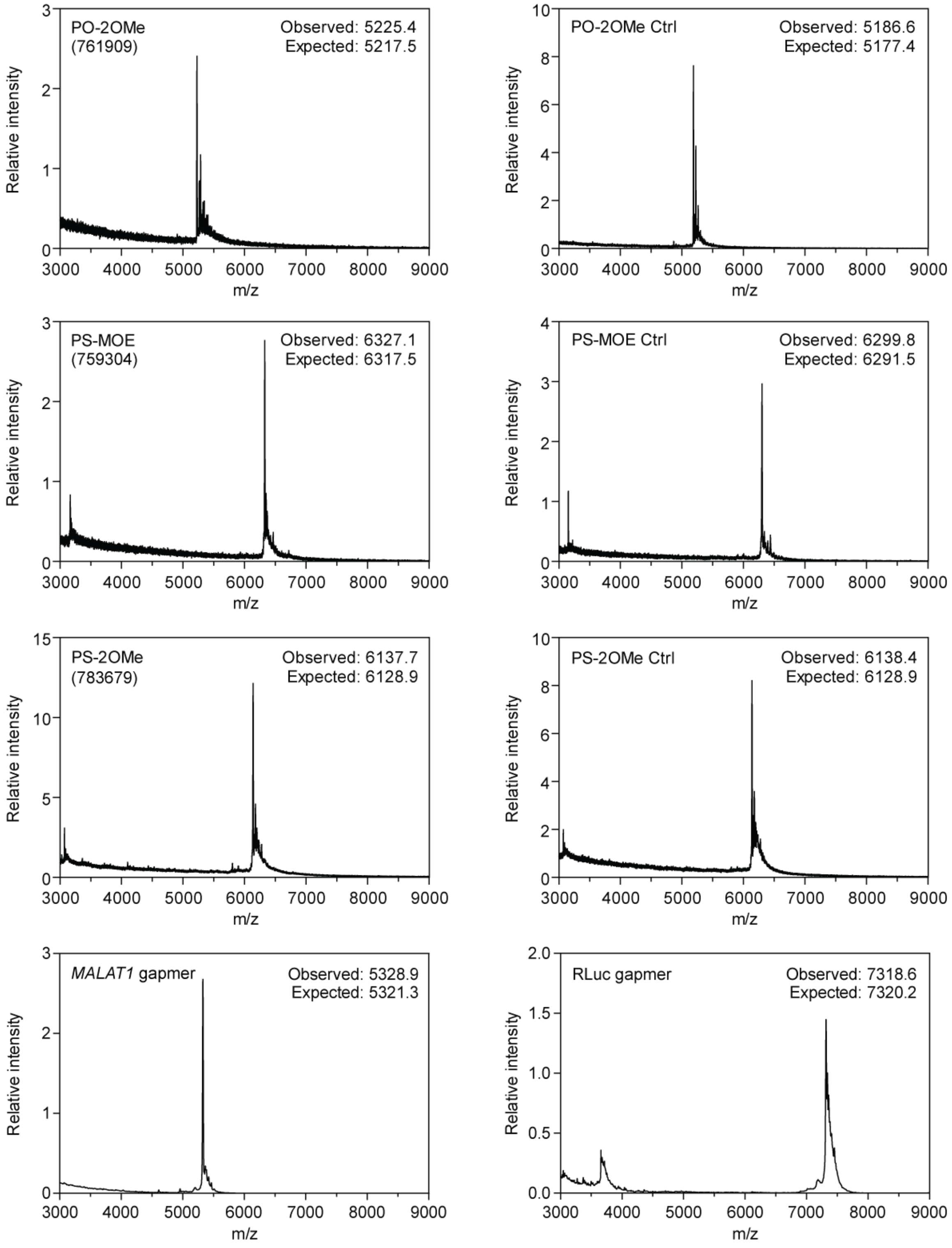
PO-2OMe
(761909)
Observed: 5225.4
Expected: 5217.5
PO-2OMe Ctrl
Observed: 5186.6
Expected: 5177.4
PS-MOE
(759304)
Observed: 6327.1
Expected: 6317.5
PS-MOE Ctrl
Observed: 6299.8
Expected: 6291.5
PS-2OMe
(783679)
Observed: 6137.7
Expected: 6128.9
PS-2OMe Ctrl
Observed: 6138.4
Expected: 6128.9
MALAT1 gapmer
Observed: 5328.9
Expected: 5321.3
RLuc gapmer
Observed: 7318.6
Expected: 7320.2
Relative intensity
m/z

Figure S6

Confirmation of oligonucleotide integrity by MALDI-TOF-MS.

MALDI-TOF-MS spectra for oligonucleotides used in this study. Observed and expected mass values are indicated.

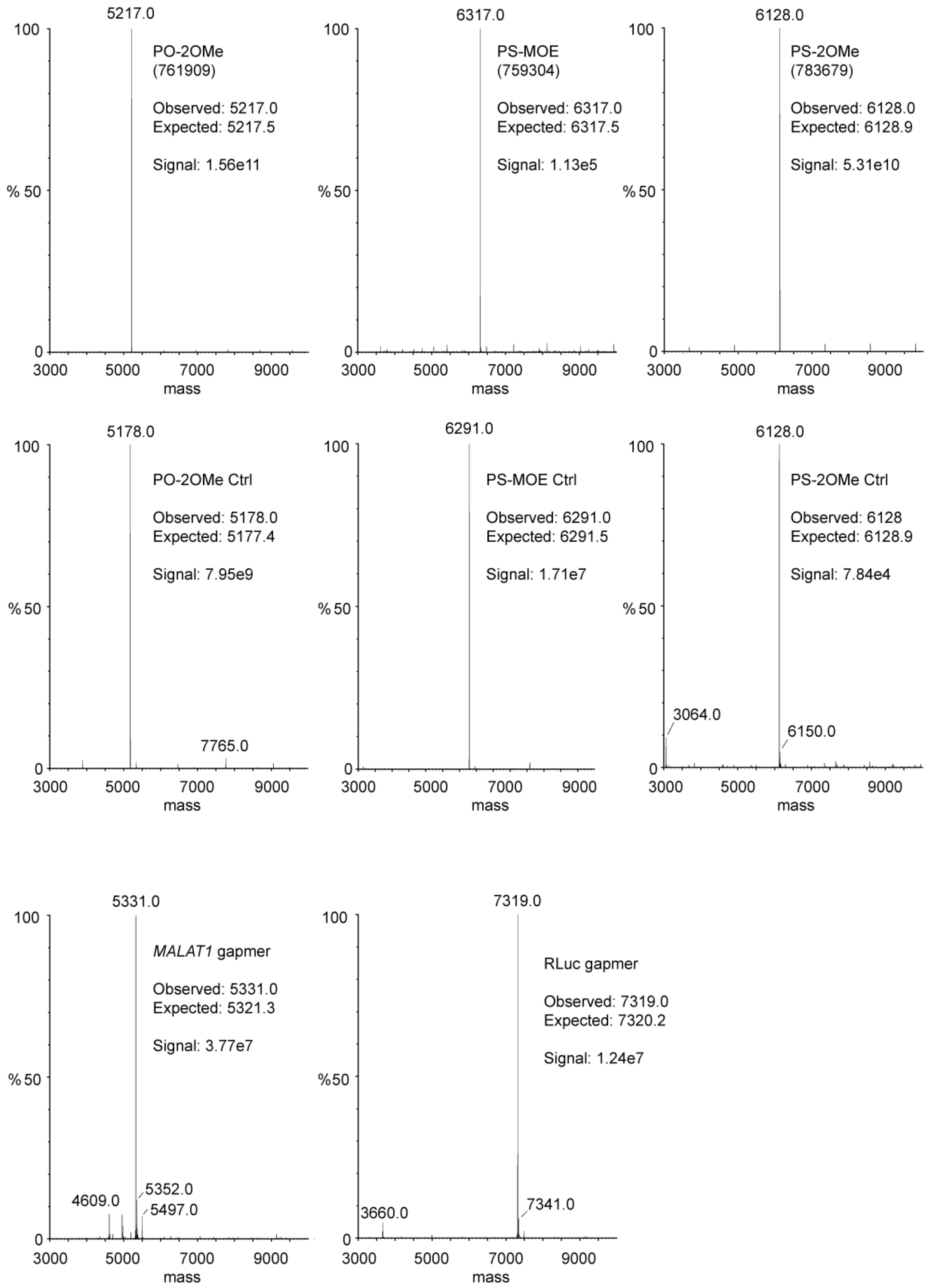

5217.0
PO-2OMe
(761909)
Observed: 5217.0
Expected: 5217.5
Signal: 1.56e11
6317.0
PS-MOE
(759304)
Observed: 6317.0
Expected: 6317.5
Signal: 1.13e5
6128.0
PS-2OMe
(783679)
Observed: 6128.0
Expected: 6128.9
Signal: 5.31e10
5178.0
PO-2OMe Ctrl
Observed: 5178.0
Expected: 5177.4
Signal: 7.95e9
7765.0
6291.0
PS-MOE Ctrl
Observed: 6291.0
Expected: 6291.5
Signal: 1.71e7
6128.0
PS-2OMe Ctrl
Observed: 6128
Expected: 6128.9
Signal: 7.84e4
3064.0
6150.0
5331.0
MALAT1 gapmer
Observed: 5331.0
Expected: 5321.3
Signal: 3.77e7
4609.0
5352.0
5497.0
7319.0
RLuc gapmer
Observed: 7319.0
Expected: 7320.2
Signal: 1.24e7
3660.0
7341.0
100
% 50
0
3000
5000
7000
9000
mass

Figure S7

Confirmation of oligonucleotide integrity by LC-MS.

Mass spectra for oligonucleotides used in this study as analysed by LC-MS. ASO samples were adjusted to 40 µM in water in a total volume of 50 µl prior to separation. Observed and expected mass values are indicated. Electrospray signal intensity is shown for each spectrum.

Table S1

Sequences of oligonucleotides used in this study.

All sequences are written 5′ to 3′. Sequences are provided in IDT notation and as the unmodified sequence only.

**RNASEH1-PO2Me (761909)**	
mCmAmUmUmUmCmGmAmCmUmCmCmCmGmGmC	CAUUUCGACUCCCGGC
**RNASEH1-PO2Me-Control**	
mCmGmCmUmUmCmAmCmUmGmCmCmUmAmCmC	CGCUUCACUGCCUACC
**RNASEH1-PSMOE (759304)**	
/52MOErC/*/i2MOErA/*/i2MOErT/*/i2MOErT/*/i2MOErT/*/i2MOErC/*/i2MOErG/*/i2MOErA/*/i2MOErC/*/i2MOErT/*/i2MOErC/*/i2MOErC/*/i2MOErC/*/i2MOErG/*/i2MOErG/*/32MOErC/	CATTTCGACTCCCGGC
**RNASEH1-PSMOE-Control**	
/52MOErC/*/i2MOErG/*/i2MOErC/*/i2MOErT/*/i2MOErT/*/i2MOErC/*/i2MOErA/*/i2MOErC/*/i2MOErT/*/i2MOErG/*/i2MOErC/*/i2MOErC/*/i2MOErT/*/i2MOErA/*/i2MOErC/*/32MOErC/	CGCTTCACTGCCTACC
**RNASEH1-PS2Me (783679)**	
mC*mA*mU*mU*mU*mC*mG*mA*mC*mU*mC*mC*mC*mG*mG*mC*mC*mC	CAUUUCGACUCCCGGCCC
**RNASEH1-PS2Me-Control**	
mC*mG*mC*mU*mU*mC*mA*mC*mU*mG*mC*mC*mU*mA*mC*mC*mG*mC	CGCUUCACUGCCUACCGC
***MALAT1* gapmer**	
+C*+T*+A*G*T*T*C*A*C*T*G*A*A*+T*+G*+C	CTAGTTCACTGAATGC
**RLuc gapmer**	
/52MOErT/*/i2MOErG/*/i2MOErT/*/i2MOErA/*/i2MOErG/*G*A*G*T*A*G*T*G*A*A*/i2MOErA/*/i2MOErG/*/i2MOErG/*/i2MOErC/*/32MOErC/	TGTAGGAGTAGTGAAAGGCC

Table S2

RT-qPCR assays used in this study.

All sequences are written 5′ to 3′.

Target	Forward	Reverse	Efficiency
RNASEH1	GCAGACAAACCAAAGAGCG	TCCAACCTTGAACCCAGTTAG	1.8240
MALAT1	GCGTAATGGAAAGTAAAGCCC	CAAACACCTCACAAAACCCC	1.8265
RPL10	CCTCTTTCCCTTCGGTGTG	AATCTTGGCATCAGGGACAC	1.8030
**RLuc**	GTAACGCTGCCTCCAGCTAC	CCAAGCGGTGAGGTACTTGT	1.6530
**FLuc**	ACTCTAAGACCGACTACCAGG	GTAGACCCAGAGCTGTTCATG	1.8110
