# Supplementary material for: uORF-targeting steric block antisense oligonucleotides do not reproducibly increase RNASEH1 expression
Source: Mol Ther Nucleic Acids. 2024 Nov 28;36(1):102406. doi: 10.1016/j.omtn.2024.102406 (PMC11697566; doi:10.1016/j.omtn.2024.102406)
Supplement: Document S2. Article plus supplemental information [file mmc2.pdf]

Molecular Therapy
Nucleic Acids
Original Article

uORF-targeting steric block antisense oligonucleotides do not reproducibly increase RNASEH1 expression

Nina Ahlskog,[1,2] Nenad Svrzikapa,[1,3,4] Rushdie Abuhamdah,[1,2] Mahnseok Kye,[1,2] Yahya Jad,[1,2] Ning Feng,[1,2] Britt Hanson,[1,5] Matthew J.A. Wood,[1,2,6] and Thomas C. Roberts[1,2,6]

[1]Department of Paediatrics, University of Oxford, Headington, Oxford OX3 7TY, UK; [2]Institute of Developmental and Regenerative Medicine, University of Oxford, IMS-Tetsuya Nakamura Building, Old Road Campus, Roosevelt Drive, Headington, Oxford OX3 7TY, UK; [3]Wave Life Science, Cambridge, MA 02138, USA; [4]Orfonyx Bio Ltd., BioEscalator, University of Oxford, Innovation Building, Rm. 10.15, Roosevelt Drive, Oxford OX3 7FZ, UK; [5]Department of Physiology, Anatomy, and Genetics, University of Oxford, South Parks Road, Oxford OX1 3PT, UK; [6]MDUK Oxford Neuromuscular Centre, South Parks Road, Oxford OX3 7TY, UK

Upstream open reading frames (uORFs) are *cis*-regulatory motifs that are predicted to occur in the 5′ UTRs of the majority of human protein-coding transcripts and are typically associated with translational repression of the downstream primary open reading frame (pORF). Interference with uORF activity provides a potential mechanism for targeted upregulation of the expression of specific transcripts. It was previously reported that steric block antisense oligonucleotides (ASOs) can bind to and mask uORF start codons to inhibit translation initiation, and thereby disrupt uORF-mediated gene regulation. Given the relative maturity of the oligonucleotide field, such a uORF blocking mechanism might have widespread therapeutic utility. Here, we re-synthesized three of the most potent ASOs targeting the *RNASEH1* uORF described in a study by Liang et al. and investigated their potential for RNASEH1 protein upregulation, with care taken to replicate the conditions of the original study. No upregulation (of endogenous or reporter protein expression) was observed with any of the oligonucleotides tested at doses ranging from 25 to 300 nM. Conversely, we observed downregulation of expression in some instances. We conclude that previously described *RNASEH1* uORF-targeting steric block ASOs are incapable of upregulating pORF protein expression in our hands.

INTRODUCTION

Steric block oligonucleotides are short single-stranded nucleic acid polymers that bind to target nucleic acid molecules via Watson-Crick base pairing in order to interfere with some binding partner interaction. Multiple steric block antisense oligonucleotides (ASOs) have now received regulatory approval for the treatment of Duchenne muscular dystrophy (eteplirsen, viltolarsen, golodirsen, and casimersen) and spinal muscular atrophy (nusinersen), with further approvals likely for these indications and others.[1] Decades of pre-clinical and clinical development have established patterns of ASO chemical modification and routes of delivery that provide blueprints for the generation of new therapeutics. The promise of such molecular medicines is that by careful sequence design, successful ASO platform chemistries can be directed to different gene targets. This is exemplified by milasen, a steric block ASO (based on the nusinersen template) designed to treat a single patient with neuronal ceroid lipofuscinosis 7.[2] Steric block ASOs have been used primarily for splice correction (i.e., to induce exon skipping or exon inclusion so as to correct the translation reading frame in otherwise out-of-frame transcripts). However, steric block oligonucleotides have similarly been utilized for multiple other purposes, including splice corruption to disrupt the translation reading frame,[3] generation of ectopic proteins with novel functions,[4] to target polyadenylation signals for modulating differential poly(A) tailing,[5] to disrupt exon-junction complex formation to relieve nonsense-mediated decay,[6] removal of "poison" exons containing premature termination codons (also known as targeted augmentation of nuclear gene expression),[6–8] inhibition of translation initiation for gene silencing,[9,10] and to target upstream open reading frames (uORFs).[11]

uORFs consist of a start codon located within the 5′ UTR of mRNA, followed by an in-frame stop codon. uORFs are common in mammalian transcripts and are typically associated with translational repression of the downstream primary ORF (pORF).[12] In 2016, a team from Ionis Pharmaceuticals (Liang et al.) reported the activation of four genes (human: *RNASEH1*, *SFXN3*, and murine: *Mrpl11*, *Lrpprc*) using a variety of uORF-targeting steric block ASOs.[11] There is currently a paucity of technologies capable of targeted activation of specific genes. As such, the Liang et al. study was important, as it suggested that relief of uORF-mediated repression could be utilized as a widely applicable means of therapeutic gene upregulation. To this end, we were motivated to explore the potential of uORF-targeting ASOs. Here, we

Received 3 August 2024; accepted 25 November 2024;
https://doi.org/10.1016/j.omtn.2024.102406.

Correspondence: Thomas C. Roberts, Institute of Developmental and Regenerative Medicine, University of Oxford, IMS-Tetsuya Nakamura Building, Old Road Campus, Roosevelt Drive, Headington, Oxford OX3 7TY, UK.
E-mail: thomas.roberts@idrm.ox.ac.uk

Published by Elsevier Inc. on behalf of The American Society of Gene and Cell Therapy.
This is an open access article under the CC BY license (http://creativecommons.org/licenses/by/4.0/).

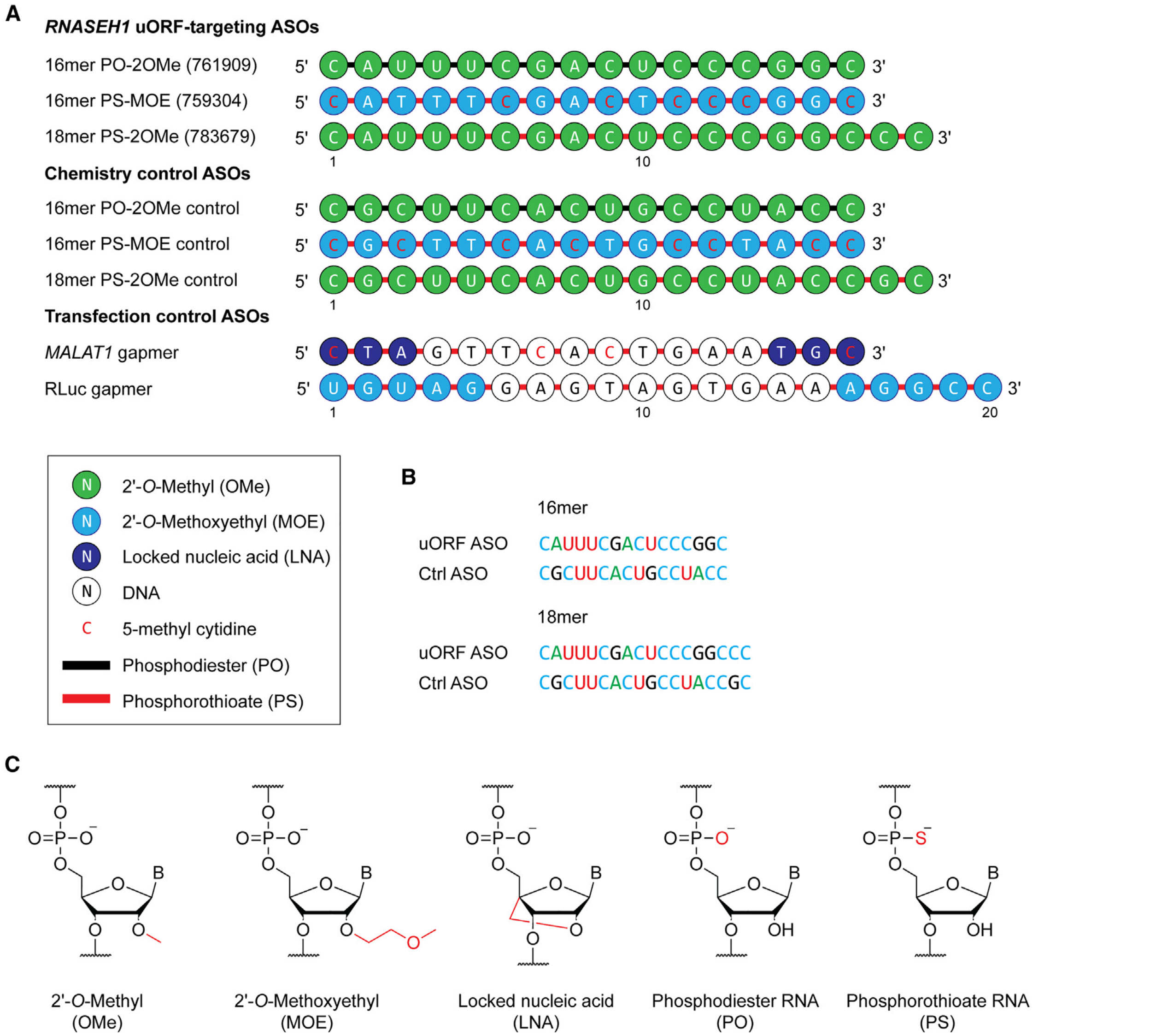

Figure 1. *RNASEH1* uORF-targeting steric block antisense oligonucleotides

(A) Schematic of ASO sequences and chemistries used in this study. (B) Sequences of on-target *RNASEH1* uORF-targeting ASOs and their scrambled control (Ctrl) sequences for the 16mer and 18mer variants. (C) Chemical structures of key oligonucleotide modifications incorporated into ASOs used in this study.

report extensive efforts to reproduce the findings reported by Liang et al.[11] We conclude that previously described steric block ASOs do not reproducibly activate RNASEH1 (ribonuclease H1) expression via uORF start codon masking.

RESULTS

uORF-targeting ASOs

We selected three oligonucleotides that exhibited the highest degree of RNASEH1 protein upregulation, as reported by Liang et al.[11] These molecules consisted of (1) a 16mer phosphodiester 2′-*O*-methyl RNA (PO-2OMe), (2) a 16mer phosphorothioate 2′-*O*-methoxyethyl RNA (PS-MOE), and (3) an 18mer phosphorothioate 2′-*O*-methyl RNA (PS-2OMe) (Figure 1A). These ASOs are referred to as 761909, 759304, and 783679, respectively, in the Liang et al. study.[11] These oligonucleotides consisted of the same sequence (with the exception of the 18mer, which included an additional two nucleotides at the 3′ terminus). Chemistry controls were synthesized in parallel (Figure 1A), in which the constituent nucleotide sequence was scrambled while the patterns of each chemistry were maintained (Figure 1B). A gapmer ASO targeting the long non-coding RNA (lncRNA) *MALAT1*[13,14] was synthesized as a control for transfection. This ASO consisted of a 3-10-3 design with a fully phosphorothioate-modified backbone, locked nucleic acid flanks, and a DNA “gap” (Figure 1A). The chemical structures of modified nucleotides included in

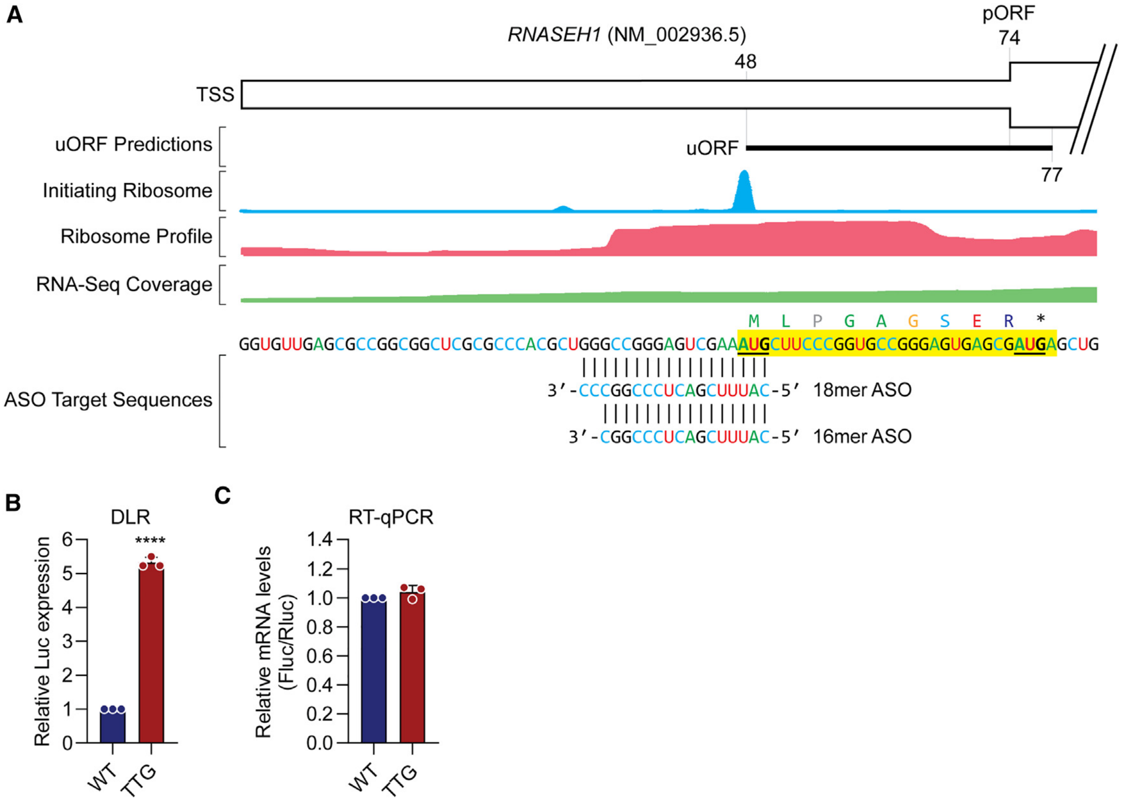

Figure 2. Validation of *RNASEH1* uORF activity

(A) Schematic of the *RNASEH1* transcript showing the position, sequence, and translation of the uORF and binding locations for the uORF-targeting ASOs. Aggregated Ribo-Seq/RNA-Seq data are overlaid, providing evidence of uORF translation. The uORF is highlighted in yellow, and the start codons are highlighted in bold and underlined. TSS, transcription start site. HEK293T cells were transfected with plasmids expressing either the wild-type (WT) *RNASEH1* 5′ UTR dual luciferase reporter (DLR) construct, or a mutant construct (TTG) in which the predicted uORF was disrupted by mutation of the uORF start codon. Treated cultures were analyzed after 24 h for (B) DLR assay or (C) RT-qPCR. Values are mean + SD (n = 3 independent experiments) and were scaled such that the mean of the WT control group was returned to a value of 1. Statistical significance was determined by unpaired Student's t test; ****$p < 0.0001$.

ASOs used in this study are shown in Figure 1C. The target *RNASEH1* transcript (NM_002936.5) contains one uORF that overlaps with the pORF and encodes a 9-amino acid peptide (Figure 2A). The 5′ ends of the uORF-targeting ASO sequences are complementary to the start codon of the *RNASEH1* uORF (Figure 2A). Publicly available ribosome sequencing (Ribo-Seq) data[15] showed a footprint of ribosome occupancy at the *RNASEH1* uORF, and a pronounced initiating ribosome peak at the corresponding upstream ATG (Figure 2A). The relative lack of ribosome initiation at the pORF ATG suggests that *RNASEH1* is likely subject to uORF-mediated translational repression.

Dual luciferase reporter (DLR) constructs were generated in which the *RNASEH1* 5′ UTR was cloned upstream of a Renilla luciferase transgene. A mutant construct in which the *RNASEH1* uORF start codon was ablated (by mutating its ATG start codon to TTG) was generated in parallel. These constructs were transfected into HEK293T cells, and relative luciferase activity was determined 24 h later. A pronounced increase (>5-fold, $p < 0.0001$) in Renilla luciferase signal was observed for the mutant TTG construct, thereby validating that the *RNASEH1* uORF does indeed repress its downstream pORF (Figure 2B), consistent with findings reported by Liang et al.[11] Changes in luciferase reproter expression could not be explained by changes in transcript levels (Figure 2C). These data suggest that this sequence is a bona fide uORF.

RNASEH1 uORF-targeting ASOs do not activate endogenous protein expression

We next sought to determine whether uORF-targeting ASOs could influence endogenous RNASEH1 protein expression. HeLa cells were transfected with 100 nM of each of the uORF-targeting ASOs (or chemistry controls), and protein was harvested over a range of time points. RNASEH1 protein upregulation effects were previously reported at 5, 10, 12, 16, and 24 h post-transfection by Liang et al.[11] We expanded this range by collecting protein lysates at 4, 8, 12, 24, 48, and 72 h post-transfection. Protein lysates were analyzed using the Jess capillary western system and a commercially available anti-RNASEH1 antibody. (Notably, the anti-RNASEH1 antibody used

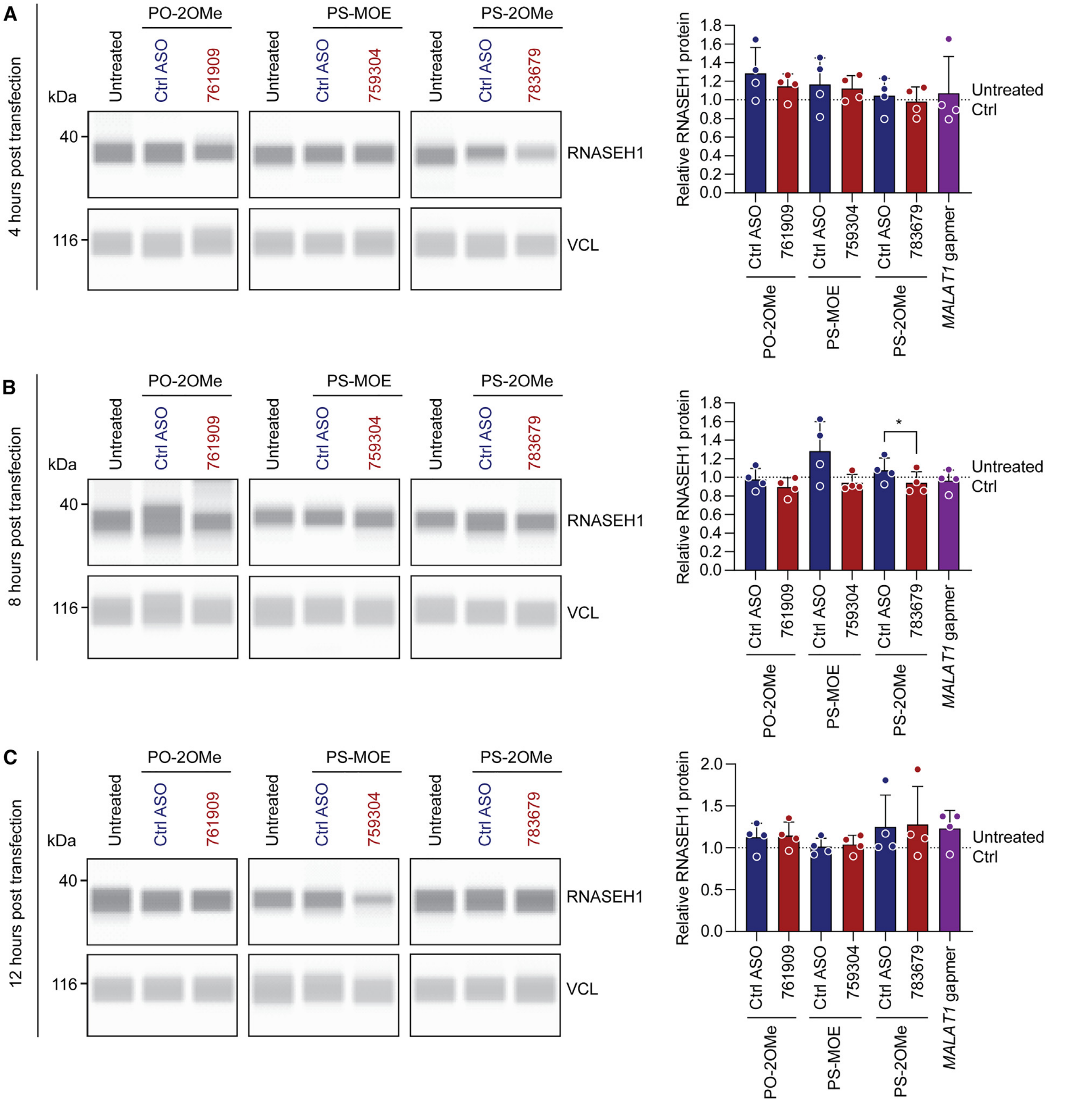

Figure 3. uORF-targeting steric block ASOs do not increase RNASEH1 protein expression at 4, 8, and 12 h post-transfection

HeLa cells were transfected with 100 nM ASOs or matched chemistry controls and cells harvested at (A) 4 h, (B) 8 h, or (C) 12 h post-transfection. RNASEH1 protein was quantified by Jess capillary western blot. Vinculin (VCL) was used as a loading control. Representative blots are shown together with histograms of protein quantification. The values of untreated control samples are indicated by dotted lines (scaled to a value of 1). A gapmer targeting *MALAT1* was included as a positive control for transfection, which is not expected to influence RNASEH1 expression. Values are mean + SD. Statistical significance was assessed by paired Student's t test between each treatment and its respective control ASO; $*p < 0.05$; $n = 4$ completely independent experiments.

in the Liang et al. study was developed in-house and thus was not available to us). Vinculin (VCL) was used as a loading control, which was highly consistent between samples. No increase in RNASEH1 protein expression was observed for any of the ASO treatments relative to either untreated cells or matched chemistry controls at any time point for $n = 4$ completely independent experiments (Figures 3 and 4). RNASEH1 western blot signal was observed at the expected size (32 kDa, 268 amino acids), and the specificity of the

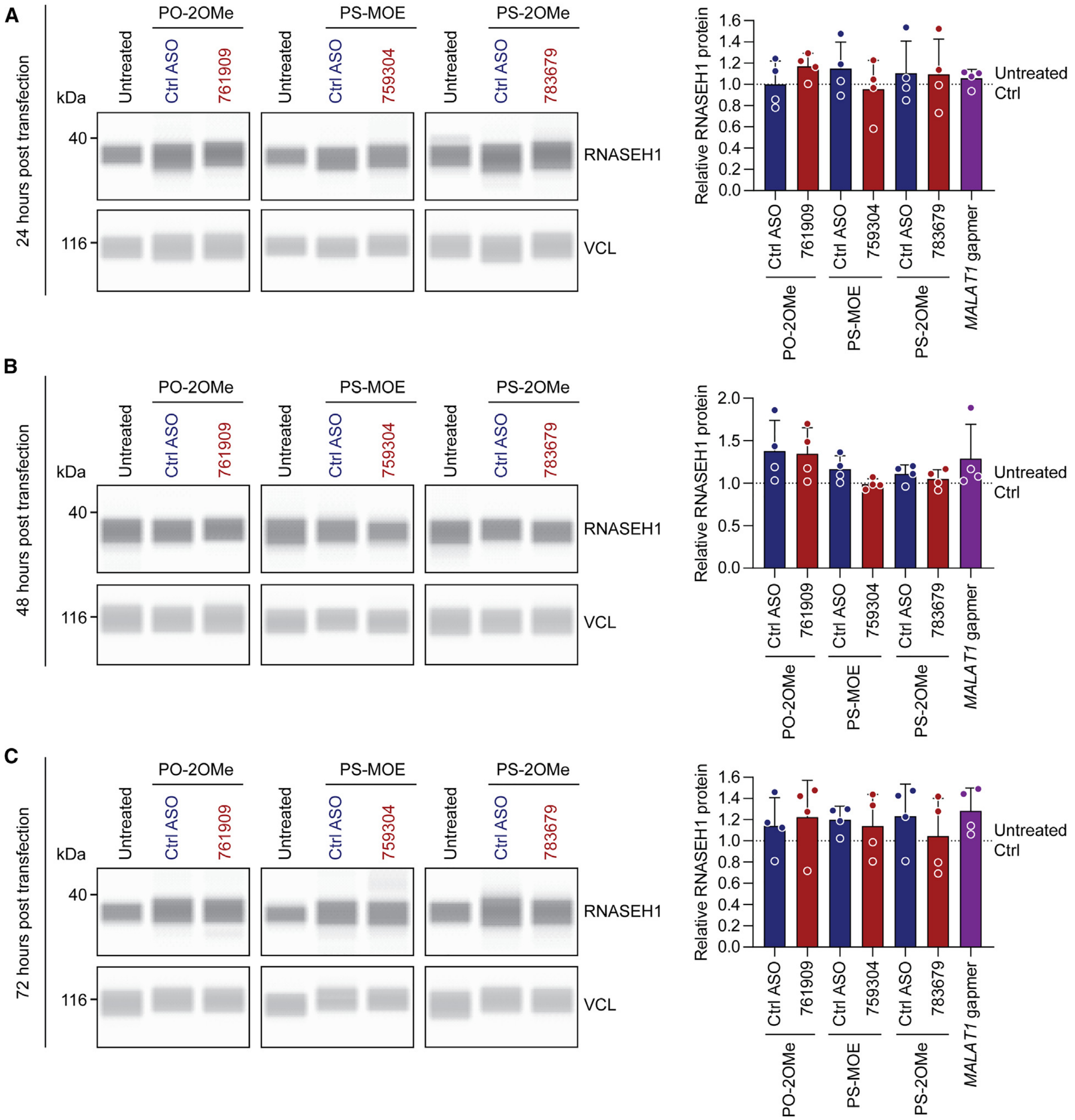

Figure 4. uORF-targeting steric block ASOs do not increase RNASEH1 protein expression at 24, 48, and 72 h post-transfection

HeLa cells were transfected with 100 nM ASOs or matched chemistry controls and cells harvested at (A) 24 h, (B) 48 h, or (C) 72 h post-transfection. RNASEH1 protein was quantified by Jess capillary western blot. VCL was used as a loading control. Representative blots are shown together with histograms of protein quantification. The value of untreated control samples is indicated by the dotted line (scaled to a value of 1). A gapmer targeting *MALAT1* was included as a positive control for transfection, which is not expected to influence RNASEH1 expression. Values are mean + SD. Statistical significance was assessed by paired Student's t test between each treatment and its respective control ASO (no significant changes detected); n = 4 completely independent experiments.

anti-RNASEH1 antibody was confirmed by small interfering RNA (siRNA) knockdown (Figure S1). Very similar data were obtained using conventional SDS-PAGE western blots for the 24- and 48-h post-transfection lysates, whereby equal protein loading was assessed by both VCL immunoblotting and Fast Green membrane staining for total protein loading (Figure S2). Parallel transfection of an ASO gapmer targeting the ubiquitously expressed lncRNA *MALAT1* resulted in target knockdown that was statistically significant

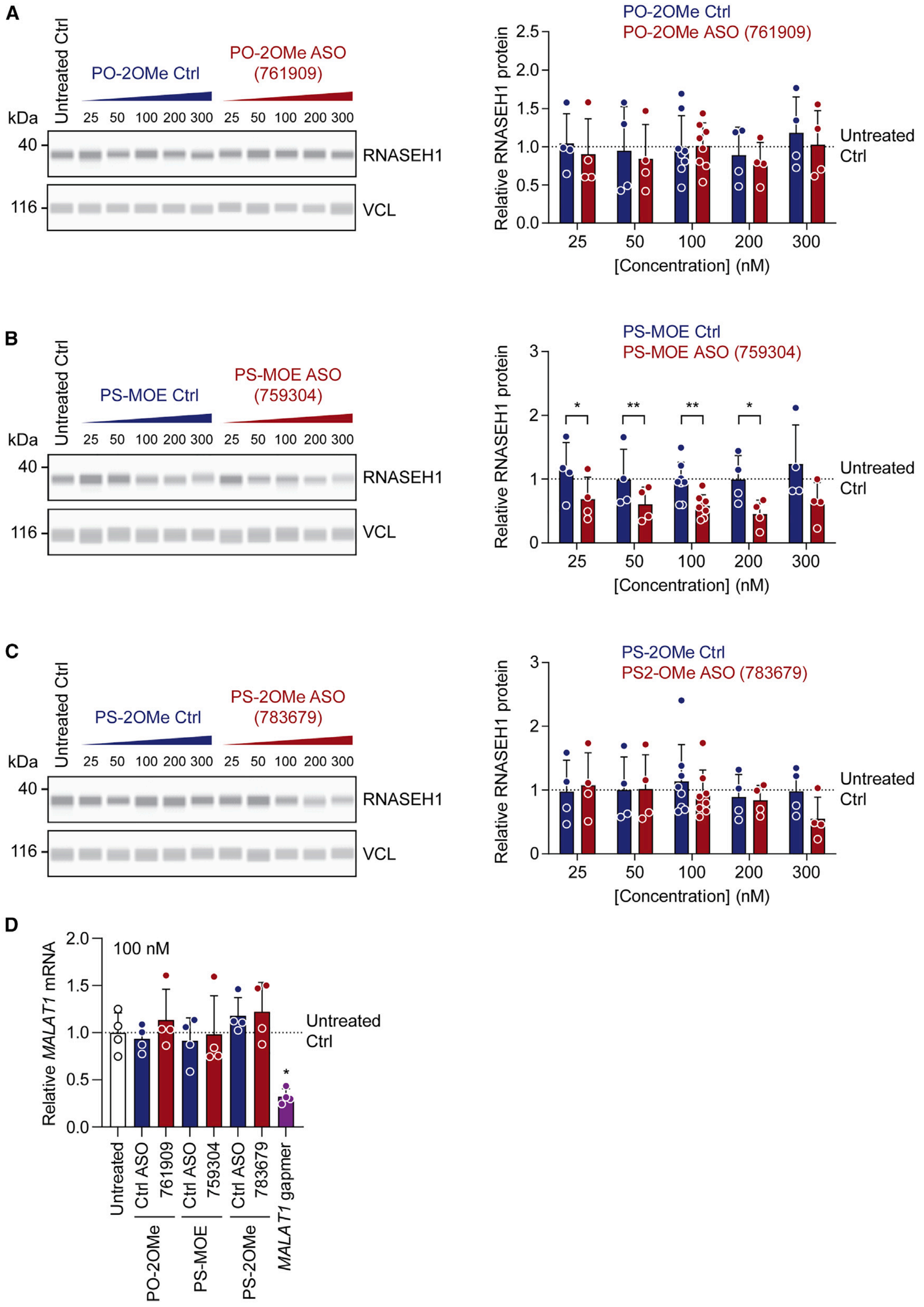

(legend on next page)

($p < 0.05$) at 24 h post-transfection. This reached a peak of >75% target knockdown relative to untreated control cultures at 72 h post-transfection ($p < 0.0001$), thereby excluding poor transfection as an explanation for a lack of RNASEH1 activation effect (Figure S3).

RNASEH1 mRNA expression levels were determined by RT-qPCR in parallel for cells collected at 24, 48, and 72 h post-transfection, whereby no statistically significant changes were observed (Figure S4). These data show that the *RNASEH1* uORF-targeting ASOs do not affect *RNASEH1* mRNA levels in these experiments.

RNASEH1 uORF-targeting ASOs do not activate endogenous protein expression at doses ranging from 25 to 300 nM

Protein upregulation effects were reported at concentrations ranging from 20 to 100 nM by Liang et al.[11] Interestingly, the same study reported parabolic dose responses in some cases or no dose response in others.[11] As such, we reasoned that potential protein upregulation responses might be observed at some concentrations but not at others. We therefore conducted a dose-response experiment in HeLa cells with all three uORF-targeting ASO chemistries (and their matched chemistry controls) at 25, 50, 100, 200, and 300 nM. Protein lysates were harvested 48 h post-transfection. No upregulation was observed for any oligonucleotide chemistry at any concentration tested (Figure 5). By contrast, treatment with PS-MOE ASOs resulted in RNASEH1 downregulation, which reached statistical significance at the 25, 50, 100, and 200 nM concentrations (Figure 5B). Treatment with the PS-2OMe ASO also reduced RNASEH1 protein expression at the 300 nM concentration, although this effect did not reach statistical significance at the $p < 0.05$ level. Results were obtained for $n = 4$ completely independent experiments (and $n = 8$ independent experiments for the 100 nM concentration). The validity of the transfection protocol was confirmed for these experiments using the *MALAT1*-targeting ASO gapmer, which exhibited ∼75% knockdown relative to untreated controls, and all other non-MALAT1-targeting ASOs (Figure 5D, $p < 0.001$).

RNASEH1 mRNA expression levels were determined by RT-qPCR in parallel (Figure S5). For PO-2OMe and PS-2OMe ASOs, there were no differences between on-target and matched chemistry controls (Figure S5A and S5C). For PS-MOE ASOs, small increases in *RNASEH1* expression were observed for the on-target ASOs, which only reached statistical significance at the $p < 0.05$ level for the 100 nM treatment group (Figure S5B). This observation is in stark contrast to the protein level downregulation observed with these oligonucleotides (Figure 5B). Together, these data show that the *RNASEH1* uORF-targeting ASOs are not reducing target transcript levels for any of the chemistries used. For the PS-MOE ASOs, the observed protein level downregulation cannot be explained by changes in mRNA expression.

uORF-targeting ASOs do not activate *RNASEH1* 5′ UTR luciferase reporters

Liang et al. previously reported that uORF-targeting ASOs can activate luciferase reporter constructs.[11] We therefore transfected HeLa cells with *RNASEH1* 5′ UTR-DLR reporter plasmids, followed by a second transfection with uORF-targeted ASOs and luciferase activity determined 24 h later (Figure 6A). ASOs were transfected at final concentrations of 100 or 50 nM together with matched chemistry controls. An ASO gapmer targeting RLuc was utilized as a positive control for transfection. This ASO consisted of a 5-10-5 design with a fully phosphorothioate-modified backbone, MOE flanks, and a DNA gap (Figure 1A). Transfection of the *RNASEH1*-TTG mutant plasmid (with the uORF start codon disrupted) was included as an additional control intended to demonstrate the theoretical maximum RNASEH1 upregulation effect. No significant changes were observed for any uORF-targeting ASO relative to the untreated control, or to any of the matched chemistry controls at either dose (Figure 6B). The positive control RLuc gapmer significantly ($p < 0.05$) reduced target expression by ∼66%, indicative of a robust dual transfection protocol (Figure 6B; $n = 5$ independent experiments). Very similar results were observed when the same experiment was performed in HEK293T cells (Figure 6C; $n = 3$ independent experiments). These data suggest that previously described uORF-targeting steric block ASOs do not activate *RNASEH1* 5′ UTR reporter constructs.

Confirmation of ASO integrity

We performed a series of analyses to exclude the possibility that the integrity of ASOs used in the present study had somehow been compromised. The integrity of each ASO was assessed by MALDI-TOF-mass spectrometry (MS), whereby prominent, single *m/z* peaks were observed for all oligonucleotides (with double-ionized peaks also detected in some instances) (Figure S6). The observed *m/z* peak values for each ASO were within less than 0.2% of the expected mass. Similarly, ASOs were analyzed by liquid chromatography-MS (LC-MS), whereby prominent single peaks were observed for each oligonucleotide with an observed mass within 0.2% of the expected mass (and all but one ASO was within >0.02%) (Figure S7). These data confirm the integrity of the ASOs used in this study.

DISCUSSION

Using the three most potent uORF-targeting steric block oligonucleotides described by Liang et al.,[11] we were unable to observe an

Figure 5. uORF-targeting steric block ASOs do not increase RNASEH1 protein expression, regardless of dose
HeLa cells were transfected with ASOs at concentrations as indicated and protein harvested after 48 h for (A) PO-2OMe, (B) PS-MOE, and (C) PS-2OMe nucleic acid chemistries. RNASEH1 protein was quantified by Jess capillary western blot. VCL was used as a loading control. Representative blots are shown together with histograms of protein quantification. The value of untreated control samples is indicated by the dotted line (scaled to a value of 1). (D) Cells were transfected with a gapmer targeting *MALAT1* (100 nM) in parallel as a positive control for transfection. *MALAT1* transcript levels were determined by RT-qPCR and normalized to *RPL10* expression. Values are mean + SD. Statistical significance for protein data were assessed by paired Student's t test within each oligonucleotide dose. RT-qPCR data for *MALAT1* expression were analyzed by one-way ANOVA and Tukey post hoc test. *$p < 0.05$; **$p < 0.01$; ***$p < 0.001$; $n = 4$ or 8 independent experiments as indicated.

increase in RNASEH1 protein relative to untreated cultures or cells treated with matched chemistry controls. This was true irrespective of model system, ASO chemistry, ASO length, ASO dose, or collection time point (Figures 3, 4, 5, and 6). The RNASEH1 antibody was validated as specific (Figure S1), and protein quantification was performed using both the Jess capillary western blot system and standard SDS-PAGE western blots (Figures 3, 4, 5, and S2). No significant changes in *RNASEH1* transcript levels were observed for ASO-treated cultures that would explain these data (Figures S4 and S5). uORF-targeting ASOs were synthesized twice, using a commercial supplier (Integrated DNA Technologies [IDT]). Successful ASO transfection was confirmed by simultaneous treatment with a gapmer targeting *MALAT1* (Figures 5D and S3). The integrity of ASOs was confirmed by in-house MALDI-TOF-MS and LC-MS analyses (Figures S6 and S7). Based on these experiments, we conclude that we are unable to reproduce the findings of Liang et al. with respect to the RNASEH1 upregulation reported previously.[11]

Steric block ASOs (and specifically, phosphorodiamidate morpholino oligonucleotides) have been used extensively to target the 5′ UTRs (usually, but not exclusively, in the vicinity of the pORF start codon) as a gene silencing technology, especially in the zebrafish *Danio rerio*.[9,10,16–18] Such translation-blocking ASOs have been assessed in clinical trials, as in the case of AVI-4126 for the targeting of the *MYC* oncogene.[19] In these cases, ASO binding is expected to interfere with the assembly of the 80S ribosome and/or prevent ribosome procession. Whether the 40S subunit can continue scanning beyond the site of ASO binding is unknown. However, were that to be the case, then targeting sequences in the 5′ UTR (i.e., uORFs) with ASOs might be expected to inhibit both uORF and pORF translation, with the net effect being translational repression of the pORF. Such a mechanism would be consistent with our experimental observations using the PS-MOE ASO chemistry, which induced RNASEH1 downregulation instead of the intended activation (Figure 5B).

At the time of writing (May 2024), the study by Liang et al. published in *Nature Biotechnology*[11] has been cited 173 times. A follow-up study by the same authors was published in 2017 in *Nucleic Acids Research*[20] and has been cited 95 times, with a total of 210 unique citations across both articles. The majority of citing articles were reviews (N = 105, 50%). Research articles constituted 27% of all citations (N = 56), which were further analyzed to determine the extent to which uORF-targeting ASOs have been adopted by other researchers. Three articles were identified as being particularly relevant. For example, Sasaki et al. reported the upregulation of cystic fibrosis transmembrane

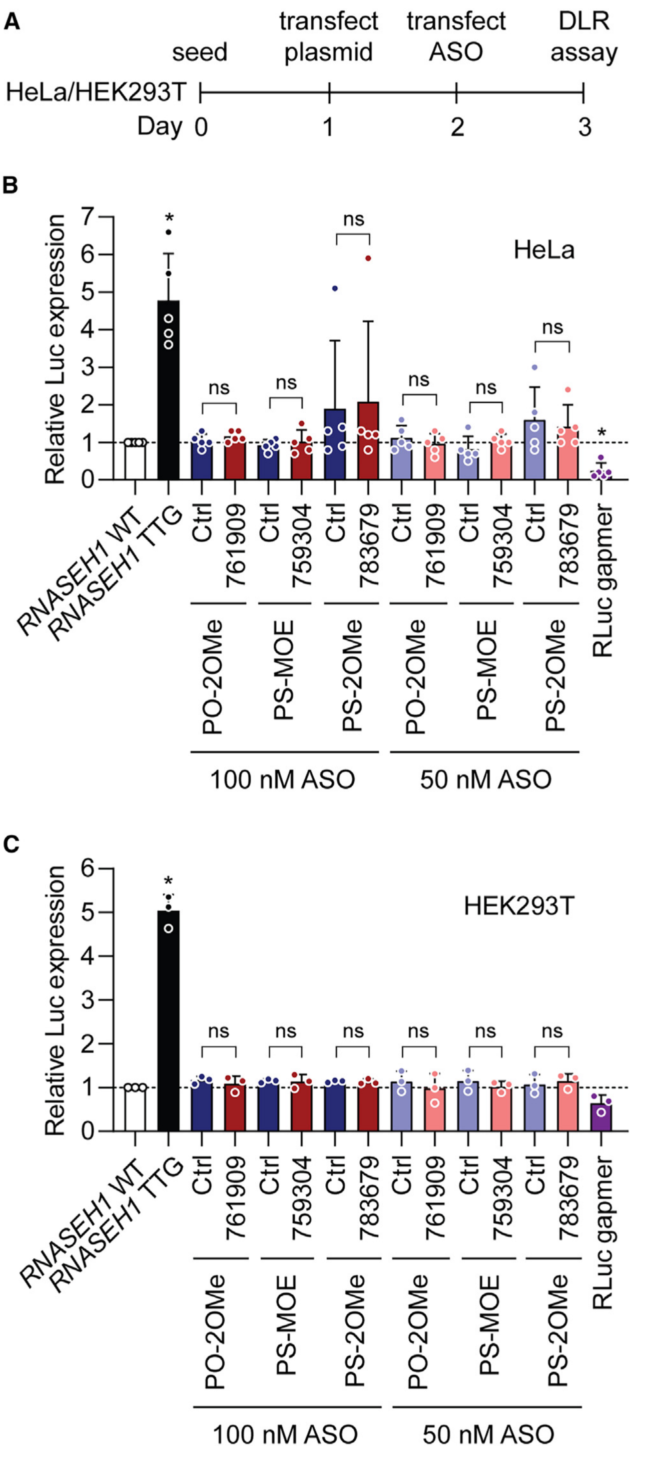

Figure 6. uORF-targeting steric block ASOs do not activate a *RNASEH1* 5′ UTR-driven luciferase reporter construct

(A) Schematic of experimental design for sequential plasmid and ASO transfection. Cells were first transfected with plasmids encoding *RNASEH1* 5′ UTR DLR constructs. After 24 h, cells were transfected with ASOs as indicated. Cells were subsequently harvested after a further 24 h. Renilla luciferase activity was determined for *RNASEH1* 5′ UTR constructs and signal normalized to firefly luciferase (encoded from a cistronically independent transgene cassette) for (B) HeLa cells (n = 5 independent experiments), and (C) HEK293T cells (n = 3 independent experiments). A gapmer targeting RLuc (100 nM) was used as a positive control for transfection. A TTG mutant in which the *RNASEH1* uORF start codon is disrupted indicated the theoretical maximum of reporter upregulation. Values are mean + SD. Statistical significance was assessed by one-way ANOVA and Tukey post hoc test, ns, not significant; *$p < 0.05$. Statistical comparisons are to the *RNASEH1* WT group unless otherwise indicated.

conductance regulator (CFTR) using uORF-targeting ASOs.[21] Furthermore, ASOs targeting a non-uORF translation repression element also induced CFTR protein upregulation. No ASO sequences were provided in this report, meaning that it is not possible to assess the exact target locations of the functional ASOs at this time. Interestingly, in this study, simultaneous disruption of the uORF start codon and a neighboring structured region was required to induce the activation of a downstream reporter gene.[21] Similarly, Kidwell et al. also reported targeting activation of murine *Ppp1r15a* using steric block ASOs, which bind to a pORF-proximal uORF.[22] In this case, a uORF start codon overlapping ASO was found to induce a modest increase in reporter expression, but an ASO that bound downstream and which did not overlap with the uORF start codon induced more robust upregulation (including for endogenous PPP1R15A protein in mouse kidney).[22] A uORF targeting strategy was also reported by Tan et al. using steric block ASOs targeting the 5′ UTR of *NUDT21* in the context of kidney renal clear cell carcinoma.[23] While robust NUDT21 protein upregulation was observed, the functional ASOs were actually non-overlapping with the predicted uORF start codon, whereas overlapping ASOs were non-functional, suggestive of a distinct mechanism.[23]

Importantly, a number of the other citing studies reported upregulation effects by targeting 5′ UTR sequences with steric block ASO oligonucleotides in a uORF-independent manner. For example, it has been reported that ASOs targeting 5′ UTR structural elements can induce targeted protein upregulation.[20] Steric block ASOs designed to disrupt the interplay between 5′ UTR double-stranded RNA motifs and uORF translation were shown to modulate pORF translation.[24] Interestingly, Hedaya et al. showed that steric block ASOs that target the uORF start codon in the 5′ UTR of *GATA4*/*Gata4* resulted in pORF repression—that is, the opposite effect to that reported by Liang et al. and consistent with our findings with PS-MOE ASOs targeting *RNASEH1* (Figure 5B).[24] By contrast, ASOs targeted to sequence surrounding the uORF start codon prevented the formation of secondary structure elements required for uORF translation at the same *GATA4* locus, leading to an enhancement in pORF activity.[24] Steric block ASOs have also been shown to promote increased expression through mRNA stabilization. Specifically, ASOs targeting the frataxin (*FXR*) 5′ UTR stabilized mRNA turnover, leading to increased transcript and protein levels in a uORF-independent manner. Interestingly, the 5′ UTR-targeting ASOs in this study bound in close proximity to the pORF start codon, which might otherwise have been expected to exhibit an inhibitory effect on translation.[25] Similarly, an ASO complementary to a predicted uORF in the *SMN2* transcript resulted in mRNA stabilization and protein upregulation, which was not associated with uORF activity.[26] These studies suggest that the 5′ UTR constitutes a promising site for potential therapeutic oligonucleotides, and that in some cases, binding to uORF sequences may be incidental. The remaining citing research articles did not utilize ASOs targeting uORFs or 5′ UTRs and thus were deemed not relevant. In summary, the number of studies reporting uORF start codon-targeting steric block ASOs activating protein expression is very limited. Notably, we are aware that groups from Roche and AstraZeneca presented preliminary data on the topic of uORF targeting for protein upregulation at the 2023 Oligonucleotide Therapeutics Society meeting.

In this study, we took care to try to reproduce the experimental conditions utilized by Liang et al. to the greatest reasonable possible extent.[11] Aside from trivial differences in the use of instruments and products, the only major difference between our studies is the antibody used to detect RNASEH1. The study by Liang et al. utilized an in-house generated primary antibody that was not available to us. As such, we have taken care to validate our commercial anti-RNASEH1 antibody by siRNA-mediated knockdown, which confirmed its suitability (Figure S1). While reproducing the upregulation effects reported for RNASEH1 has been challenging, the observation that this transcript is regulated by its corresponding uORF is robust (Figure 2). Notably, alternative methods of uORF interference may still provide means of therapeutic gene upregulation, especially for monogenic haploinsufficiency disorders such as Angelman syndrome, Dravet syndrome, and Rett syndrome. The application of alternate technologies such as RNA structure disruption,[24] synthetic internal ribosome entry site scaffolds,[27] SINEUP lncRNAs,[28] and exon skipping of uORF-containing exons[29] are exciting possibilities for future nucleic acid-based therapeutics to treat such disorders. In conclusion, this study casts doubt on the notion that steric block ASOs targeted to a uORF start codon can induce protein upregulation in the specific case of RNASEH1 and possibly to some extent in the general case.

MATERIALS AND METHODS

Oligonucleotides

ASOs were purchased from IDT (Leuven, Belgium) or synthesized in-house (i.e., for *MALAT1* and RLuc gapmer ASOs). All ASO sequences are listed in Table S1 and illustrated diagrammatically in Figure 1A.

siRNA pools were purchased from Dharmacon (Horizon Discovery, Cambridge, UK). ON-TARGETplus Human RNASEH1 siRNA (246243) (target sites: 5′-GACAGUAUGUUUACGAUAA-3′, 5′-GAGCACAGGUGGACCGGUU-3′, 5′-ACAAGAAUCGGAGGCGAAA-3′, 5′-GAGCAGGAAUCGGCGUUUA-3′). A non-targeting siRNA pool was used as a negative control (D-001810-10-05, Dharmacon).

Cell cultures

HeLa and HEK293T cells were grown in culture media composed of DMEM-GlutaMax supplemented with 10% fetal bovine serum (both Gibco, Thermo Fisher Scientific, Loughborough, UK) and 1% antibiotic/antimycotic solution (Merck Life Science, Gillingham, UK). The cells were maintained in a passively humidified incubator at 37°C, 5% CO_2. Cell cultures were confirmed free of mycoplasma contamination through monthly testing.

ASOs were transfected using Lipofectamine RNAiMAX (Invitrogen) according to the manufacturer's instructions. Plasmid DNA (100 ng per well) was transfected using Lipofectamine 2000 (Invitrogen) according to the manufacturer's instructions.

Protein quantification by capillary gel electrophoresis

Cells were plated at $\sim 4 \times 10^5$ cells/well in 2 mL culture media on 6-well plates and transfected 24 h later with ASOs as above at indicated concentrations. The treated cells were returned to the incubator for 4, 8, 12, 24, 48, or 72 h. At the specified time point, the cells were collected in 150 μL ice-cold radioimmunoprecipitation assay (RIPA) buffer (Thermo Fisher Scientific) with cOmplete EDTA-free protease inhibitor (Roche, Welwyn Garden City, UK) and sonicated 3 × 5 s using a Q500 Sonicator (Qsonica LLC, Newtown, CT) at 25% amplitude. The lysates were cleared by centrifugation at 10,000 × *g* for 5 min, and the protein concentration was determined using the DC Protein Assay (Bio-Rad Laboratories, Watford, UK) according to the manufacturer's instructions. All samples were diluted to a final concentration of 1.5 mg/mL total protein in RIPA buffer prior to loading on the Jess instrument or for SDS-PAGE.

Capillary gel electrophoresis experiments were performed using the Jess Simple Western system with a 12- to 230-kDa fluorescence separation module (both Bio-Techne, Abingdon, UK) according to the manufacturer's instructions. Primary antibodies anti-RNaseH1 (1:250, catalog no. 15606 Proteintech, Manchester, UK) and anti-VCL (1:25,000, catalog no. V9131 Sigma-Aldrich, Gillingham, UK) were used for relative quantification and loading normalization purposes, respectively. Secondary antibodies αRb-IR and αMs-NIR (both Bio-Techne, Abingdon, UK) were used as per the manufacturer's instructions (i.e., undiluted). The results were analyzed using the Compass for Simple Western software (version 6.3.0) according to manufacturer's instructions.

RT-qPCR

Total RNA was isolated from the cells of 6-well plates treated as above using the Maxwell RSC simplyRNA kit and the Maxwell RSC Instrument (both Promega, Southampton, UK) according to the manufacturer's instructions. In short, the cells were washed and collected by trypsinization. The cell pellet was then resuspended in 200 μL Maxwell Homogenization solution containing 1-thioglycerol and loaded onto prefilled Maxwell RSC cartridges and run in Maxwell RSC 16 with the appropriate program. Extracted RNA was quantified by UV spectrophotometry using a Nanodrop 2000 instrument (Thermo Fisher Scientific). cDNA was reverse transcribed from 1 μg total RNA using the High-Capacity cDNA Reverse Transcription Kit (Thermo Fisher Scientific) according to the manufacturer's instructions. PCR assay efficiencies were determined using LinRegPCR.[30]

qPCR was performed on the cDNA (diluted 1 in 5 in nuclease-free water) using the Power SYBR Green PCR Master Mix and the StepOnePlus Real-Time PCR System (both Applied Biosystems, Warrington, UK). *MALAT1* expression was normalized to *RPL10* (60S ribosomal protein L10), and the relative quantification was determined using the Pfaffl method imputing experimentally determined primer efficiencies.[31] Primer sequences and efficiencies are listed in Table S2.

DLR assay

For the luciferase reporter assay, cells were plated at $\sim 1.5 \times 10^4$ cells/well in 80 μL culture media in white-walled 96-well plates. After 24 h, the cells were transfected with 100 ng/well of reporter gene plasmid with either a wild-type or a TTG-mutated 5′ UTR for *RNASEH1* using the Lipofectamine 2000 Transfection Reagent (Thermo Fisher Scientific). On the following day (48 h), the cells were transfected with either 50 or 100 nM ASO, as described above, or 100 nM RLuc gapmer (positive control for transfection). On day 3 (72 h), the cells were assayed using the Dual-Glo Luciferase Assay System (Promega) and a Clariostar Plus plate reader (BMG Labtech, Aylesbury, UK).

Western blot (SDS-PAGE)

Protein samples (20 μg total protein per lane) were separated by SDS-PAGE using precast 10% NuPAGE Bis-Tris midi gels (Thermo Fisher Scientific). Protein was electroblotted onto polyvinylidene fluoride membranes (Merck Millipore, Watford, UK). Membranes were stained with Fast Green FCF (Sigma-Aldrich) and imaged for total protein on a ChemiDoc MP Imaging System (Bio-Rad). Membranes were blocked in Intercept PBS Blocking Buffer (Li-Cor Biotechnology, Cambridge, UK) before overnight incubation with anti-RNaseH1 (1:500, Proteintech) and anti-VCL (1:20,000, Sigma-Aldrich) antibodies in blocking buffer. Following incubation with horseradish peroxidase (HRP)-linked secondary antibody (1:500 horse anti-mouse-HRP, catalog no. 7076S or goat anti-rabbit-HRP, catalog no. 7074S; both Cell Signaling Technology, Leiden, The Netherlands), the signal was developed using Clarity Western ECL Substrate and visualized on the ChemiDoc MP Imaging System (both Bio-Rad).

MALDI-TOF-MS

For MALDI-TOF-MS analysis, 1 μL 100 μM ASO was added to 10 μL MALDI matrix solution (40 mg/mL 3-hydroxypicolinic acid, 40 mM ammonium citrate in acetonitrile/water [1:1]), and mixed thoroughly. The resulting mixture was spotted (0.5 μL) onto an MALDI target plate, dried, and then analyzed using a Shimadzu MALDI-8020 instrument (Shimadzu UK, Milton Keynes, UK).

LC-MS

LC-MS analysis was performed on a Waters SQD 2 coupled to a Waters ACQUITY UPLC system using a Waters ACQUITY Premier BEH C18 1.7-μm column (2.1 × 50 mm) (all Waters, Wilmslow, UK). ASO samples were adjusted to 40 μM in water in a 50-μL volume before LC-MS analysis in negative ionization mode. Mobile phase A consisted of: 400 mM 1,1,1,3,3,3-hexafluoro-2-propanol and 15 mM triethylamine in H_2O; mobile phase B consisted of: MeOH; the flow rate was set to 0.5 mL/min; column temperature was set to 60°C. The raw continuum data were deconvoluted to produce zero-charge mass spectra using MassLynx software (Waters).

Bioinformatics

Publicly available Ribo-Seq datasets were analyzed using custom bioinformatics tools developed to rapidly analyze uORF architecture in

any transcript of interest, in the context of translating ribosomes. Briefly, RefSeq transcript level information was combined with large-scale Ribo-Seq datasets, including 46 ribosome footprinting, 8 ribosome initiation, and 34 RNA-Seq, previously described as part of the GWIPS-viz resource.[15,32] An aggregation strategy was used to amplify the signal and visualize mapped reads in areas with low coverage. Global aggregates of the Ribo-Seq datasets were generated per genomic coordinate, and the pyBigWig library was utilized to output a single global bigwig file for each track of interest (Footprints, mRNA, Initiation). SQL queries to the University of California, Santa Cruz hg38 database were used to extract sequence features that allow for real-time recalculation and mapping of uORFs and Ribo-Seq data on an RNA isoform level.[33]

Statistical analyses

Statistical analyses were performed using GraphPad Prism version 10.1.2 (GraphPad Software, La Jolla, CA).

DATA AND CODE AVAILABILITY

All data are included in the manuscript. Raw data are available on request.

ACKNOWLEDGMENTS

This work was supported by grants from Great Ormond Street Hospital Sparks Fund/Dravet Syndrome UK (V4121) and UK MRC (TransNAT) (MR/X008029/1) (awarded to M.J.A.W. and T.C.R.), the Oxford University Press John Fell Fund and Medical Life Sciences Translational Fund (awarded to T.C.R.). The authors thank Dr. Jennifer Frommer for assistance with the LC-MS measurements.

AUTHOR CONTRIBUTIONS

T.C.R., N.S., B.H., and M.J.A.W. conceived the study. T.C.R. and M.J.A.W. supervised the work. N.A., N.S., R.A., M.K., Y.J., N.F., and B.H. performed the experimentation. T.C.R. wrote the first draft of the manuscript. All authors contributed to the final version of the manuscript.

DECLARATION OF INTERESTS

T.C.R., M.J.A.W., and B.H. have filed a patent related to a uORF-targeting ASO technology. T.C.R., M.J.A.W., N.S., and B.H. are founders and shareholders in Orfonyx Bio Ltd., a biotechnology spin-out company that aims to utilize uORF-targeting technologies for therapeutics development. N.S. is an employee of Orfonyx Bio. T.C.R. and M.J.A.W. are consultants for Orfonyx Bio.

SUPPLEMENTAL INFORMATION

Supplemental information can be found online at https://doi.org/10.1016/j.omtn.2024.102406.

REFERENCES

1. Roberts, T.C., Langer, R., and Wood, M.J.A. (2020). Advances in oligonucleotide drug delivery. Nat. Rev. Drug Discov. *19*, 673–694.
2. Kim, J., Hu, C., Moufawad El Achkar, C., Black, L.E., Douville, J., Larson, A., Pendergast, M.K., Goldkind, S.F., Lee, E.A., Kuniholm, A., et al. (2019). Patient-Customized Oligonucleotide Therapy for a Rare Genetic Disease. N. Engl. J. Med. *381*, 1644–1652.
3. Ward, A.J., Norrbom, M., Chun, S., Bennett, C.F., and Rigo, F. (2014). Nonsense-mediated decay as a terminating mechanism for antisense oligonucleotides. Nucleic Acids Res. *42*, 5871–5879.
4. Gupta, D., Orehek, S., Turunen, J., O'Donovan, L., Gait, M.J., El-Andaloussi, S., and Wood, M.J.A. (2023). Modulation of Pro-Inflammatory IL-6 Trans-Signaling Axis by Splice Switching Oligonucleotides as a Therapeutic Modality in Inflammation. Cells *12*, 2285.
5. Vickers, T.A., Wyatt, J.R., Burckin, T., Bennett, C.F., and Freier, S.M. (2001). Fully modified 2' MOE oligonucleotides redirect polyadenylation. Nucleic Acids Res. *29*, 1293–1299.
6. Nomakuchi, T.T., Rigo, F., Aznarez, I., and Krainer, A.R. (2016). Antisense oligonucleotide-directed inhibition of nonsense-mediated mRNA decay. Nat. Biotechnol. *34*, 164–166.
7. Han, Z., Chen, C., Christiansen, A., Ji, S., Lin, Q., Anumonwo, C., Liu, C., Leiser, S.C., Meena, Aznarez, I., et al. (2020). Antisense oligonucleotides increase Scn1a expression and reduce seizures and SUDEP incidence in a mouse model of Dravet syndrome. Sci. Transl. Med. *12*, eaaz6100.
8. Lim, K.H., Han, Z., Jeon, H.Y., Kach, J., Jing, E., Weyn-Vanhentenryck, S., Downs, M., Corrionero, A., Oh, R., Scharner, J., et al. (2020). Antisense oligonucleotide modulation of non-productive alternative splicing upregulates gene expression. Nat. Commun. *11*, 3501.
9. Boiziau, C., Kurfurst, R., Cazenave, C., Roig, V., Thuong, N.T., and Toulmé, J.J. (1991). Inhibition of translation initiation by antisense oligonucleotides via an RNase-H independent mechanism. Nucleic Acids Res. *19*, 1113–1119.
10. Baker, B.F., Lot, S.S., Condon, T.P., Cheng-Flournoy, S., Lesnik, E.A., Sasmor, H.M., and Bennett, C.F. (1997). 2'-O-(2-Methoxy)ethyl-modified anti-intercellular adhesion molecule 1 (ICAM-1) oligonucleotides selectively increase the ICAM-1 mRNA level and inhibit formation of the ICAM-1 translation initiation complex in human umbilical vein endothelial cells. J. Biol. Chem. *272*, 11994–12000.
11. Liang, X.-H., Shen, W., Sun, H., Migawa, M.T., Vickers, T.A., and Crooke, S.T. (2016). Translation efficiency of mRNAs is increased by antisense oligonucleotides targeting upstream open reading frames. Nat. Biotechnol. *34*, 875–880.
12. Calvo, S.E., Pagliarini, D.J., and Mootha, V.K. (2009). Upstream open reading frames cause widespread reduction of protein expression and are polymorphic among humans. Proc. Natl. Acad. Sci. USA *106*, 7507–7512.
13. Hung, G., Xiao, X., Peralta, R., Bhattacharjee, G., Murray, S., Norris, D., Guo, S., and Monia, B.P. (2013). Characterization of target mRNA reduction through in situ RNA hybridization in multiple organ systems following systemic antisense treatment in animals. Nucleic Acid Ther. *23*, 369–378.
14. Kaburagi, H., Nagata, T., Enomoto, M., Hirai, T., Ohyagi, M., Ihara, K., Yoshida-Tanaka, K., Ebihara, S., Asada, K., Yokoyama, H., et al. (2022). Systemic DNA/RNA heteroduplex oligonucleotide administration for regulating the gene expression of dorsal root ganglion and sciatic nerve. Mol. Ther. Nucleic Acids *28*, 910–919.
15. Kiniry, S.J., Michel, A.M., and Baranov, P.V. (2018). The GWIPS-viz Browser. Curr. Protoc. Bioinformatics *62*, e50.
16. Bill, B.R., Petzold, A.M., Clark, K.J., Schimmenti, L.A., and Ekker, S.C. (2009). A Primer for Morpholino Use in Zebrafish. Zebrafish *6*, 69–77.
17. Moulton, J.D. (2017). Using Morpholinos to Control Gene Expression. Curr. Protoc. Nucleic Acid Chem. *68*, 4.30.1–4.30.29.
18. Summerton, J. (1999). Morpholino antisense oligomers: the case for an RNase H-independent structural type. Biochim. Biophys. Acta *1489*, 141–158.
19. Iversen, P.L., Arora, V., Acker, A.J., Mason, D.H., and Devi, G.R. (2003). Efficacy of antisense morpholino oligomer targeted to c-myc in prostate cancer xenograft murine model and a Phase I safety study in humans. Clin. Cancer Res. *9*, 2510–2519.
20. Liang, X.H., Sun, H., Shen, W., Wang, S., Yao, J., Migawa, M.T., Bui, H.H., Damle, S.S., Riney, S., Graham, M.J., et al. (2017). Antisense oligonucleotides targeting translation inhibitory elements in 5′ UTRs can selectively increase protein levels. Nucleic Acids Res. *45*, 9528–9546.
21. Sasaki, S., Sun, R., Bui, H.H., Crosby, J.R., Monia, B.P., and Guo, S. (2019). Steric Inhibition of 5' UTR Regulatory Elements Results in Upregulation of Human CFTR. Mol. Ther. *27*, 1749–1757.
22. Kidwell, A., Yadav, S.P.S., Maier, B., Zollman, A., Ni, K., Halim, A., Janosevic, D., Myslinski, J., Syed, F., Zeng, L., et al. (2023). Translation Rescue by Targeting Ppp1r15a through Its Upstream Open Reading Frame in Sepsis-Induced Acute Kidney Injury in a Murine Model. J. Am. Soc. Nephrol. *34*, 220–240.
23. Tan, Y., Zheng, T., Su, Z., Chen, M., Chen, S., Zhang, R., Wang, R., Li, K., and Na, N. (2023). Alternative polyadenylation reprogramming of MORC2 induced by NUDT21 loss promotes KIRC carcinogenesis. JCI Insight *8*, e162893.

24. Hedaya, O.M., Venkata Subbaiah, K.C., Jiang, F., Xie, L.H., Wu, J., Khor, E.S., Zhu, M., Mathews, D.H., Proschel, C., and Yao, P. (2023). Secondary structures that regulate mRNA translation provide insights for ASO-mediated modulation of cardiac hypertrophy. Nat. Commun. *14*, 6166.

25. Li, Y., Li, J., Wang, J., Lynch, D.R., Shen, X., Corey, D.R., Parekh, D., Bhat, B., Woo, C., Cherry, J.J., et al. (2021). Targeting 3' and 5' untranslated regions with antisense oligonucleotides to stabilize frataxin mRNA and increase protein expression. Nucleic Acids Res. *49*, 11560–11574.

26. Winkelsas, A.M., Grunseich, C., Harmison, G.G., Chwalenia, K., Rinaldi, C., Hammond, S.M., Johnson, K., Bowerman, M., Arya, S., Talbot, K., et al. (2021). Targeting the 5′ untranslated region of SMN2 as a therapeutic strategy for spinal muscular atrophy. Mol. Ther. Nucleic Acids *23*, 731–742.

27. Cao, Y., Liu, H., Lu, S.S., Jones, K.A., Govind, A.P., Jeyifous, O., Simmons, C.Q., Tabatabaei, N., Green, W.N., Holder, J.L., Jr., et al. (2023). RNA-based translation activators for targeted gene upregulation. Nat. Commun. *14*, 6827.

28. Zucchelli, S., Fasolo, F., Russo, R., Cimatti, L., Patrucco, L., Takahashi, H., Jones, M.H., Santoro, C., Sblattero, D., Cotella, D., et al. (2015). SINEUPs are modular antisense long non-coding RNAs that increase synthesis of target proteins in cells. Front. Cell. Neurosci. *9*, 174.

29. Ang, Z., Paruzzo, L., Hayer, K.E., Schmidt, C., Torres Diz, M., Xu, F., Zankharia, U., Zhang, Y., Soldan, S., Zheng, S., et al. (2023). Alternative splicing of its 5′-UTR limits CD20 mRNA translation and enables resistance to CD20-directed immunotherapies. Blood *142*, 1724–1739.

30. Ramakers, C., Ruijter, J.M., Deprez, R.H.L., and Moorman, A.F.M. (2003). Assumption-free analysis of quantitative real-time polymerase chain reaction (PCR) data. Neurosci. Lett. *339*, 62–66.

31. Pfaffl, M.W. (2001). A new mathematical model for relative quantification in real-time RT-PCR. Nucleic Acids Res. *29*, e45.

32. Michel, A.M., Kiniry, S.J., O'Connor, P.B.F., Mullan, J.P., and Baranov, P.V. (2018). GWIPS-viz: 2018 update. Nucleic Acids Res. *46*, D823–D830.

33. Paladin, L., Schaeffer, M., Gaudet, P., Zahn-Zabal, M., Michel, P.A., Piovesan, D., Tosatto, S.C.E., and Bairoch, A. (2020). The Feature-Viewer: a visualization tool for positional annotations on a sequence. Bioinformatics *36*, 3244–3245.

OMTN, Volume *36*

Supplemental information

uORF-targeting steric block antisense oligonucleotides do not reproducibly increase RNASEH1 expression

Nina Ahlskog, Nenad Svrzikapa, Rushdie Abuhamdah, Mahnseok Kye, Yahya Jad, Ning Feng, Britt Hanson, Matthew J.A. Wood, and Thomas C. Roberts

Supplemental Information

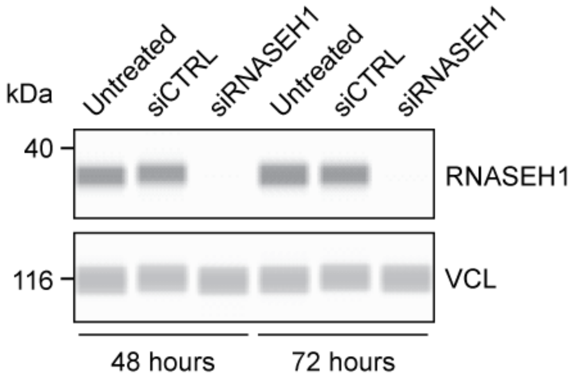

Figure S1

Anti-RNASEH1 antibody validation.

HeLa cells were transfected with a pool of siRNAs targeting RNASEH1, or a control siRNA pool and protein harvested 48 or 72 hours post transfection. RNASEH1 protein was quantified by Jess capillary western blot. Vinculin (VCL) was used as a loading control. RNASEH1 was detected at the expected size (32 kDa) and was undetectable after siRNA-mediated knockdown.

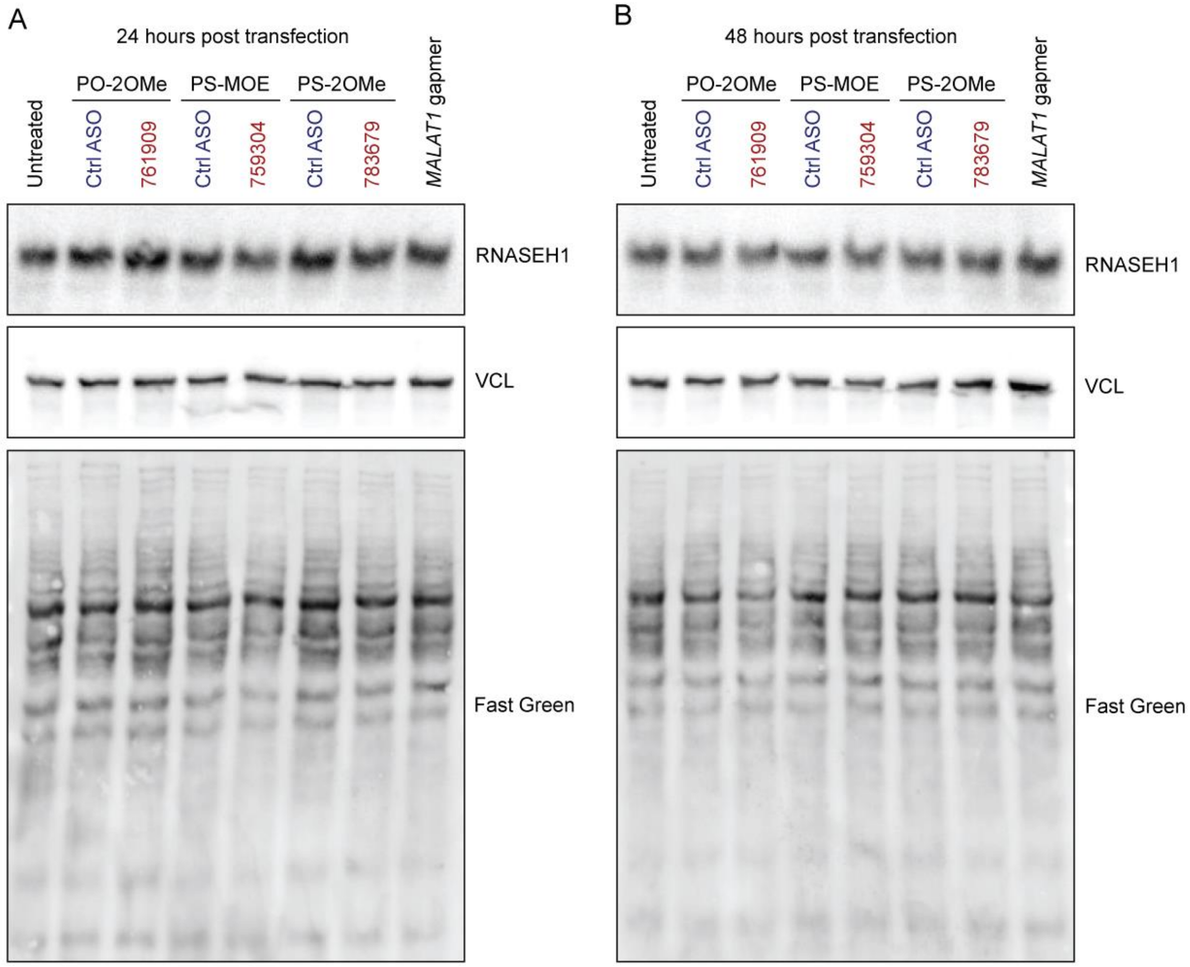

Figure S2

uORF-targeting steric block ASOs do not increase RNASEH1 protein expression at 24 and 48 hours post transfection as assessed by western blot.

HeLa cells were transfected with ASOs as indicated and protein harvested at (**A**) 24 hours, and (**B**) 48 hours post transfection. Samples were analysed by standard SDS-PAGE western blotting using anti-RNASEH1 antibodies. Vinculin (VCL) was used as a loading control protein, and total protein loading was assessed by Fast Green staining.

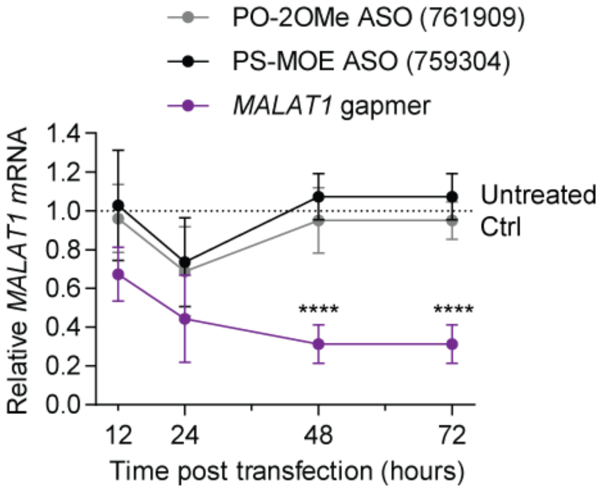

Figure S3

Validation of ASO transfection protocol.

HeLa cells were transfected with a gapmer (100 nM) ASO targeting *MALAT1* or non-MALAT1-targeting ASOs and RNA harvested at 12, 24, 48, and 72 hours post transfection. *MALAT1* transcript levels were determined by RT-qPCR and normalised to *RPL10* expression. Values are mean+SD. Untreated control samples were utilised as calibrator samples and were scaled to a value of 1 at each time point. Statistical differences were determined by one-way ANOVA and Tukey *post hoc* test performed at each time point. ***P<0.0001 (comparison of the *MALAT1* gapmer treatment versus the PS-MOE ASO), n=4 completely independent experiments.

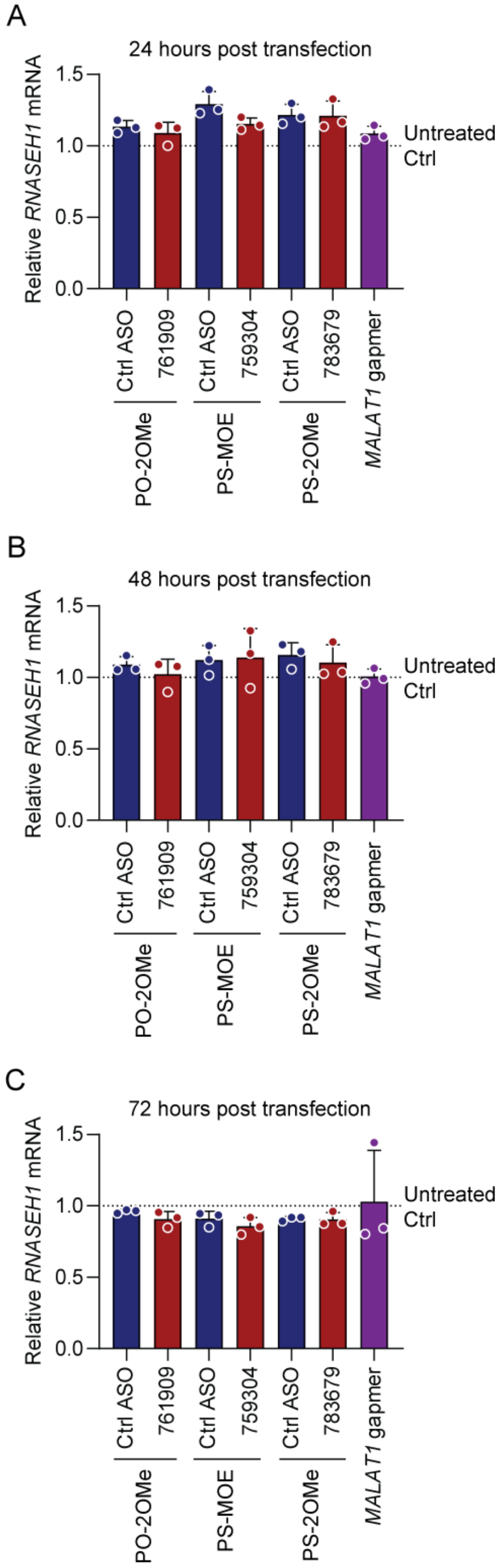
A
24 hours post transfection
Relative RNASEH1 mRNA
1.5
1.0
0.5
0.0
Untreated Ctrl
Ctrl ASO
761909
Ctrl ASO
759304
Ctrl ASO
783679
MALAT1 gapmer
PO-2OMe
PS-MOE
PS-2OMe
B
48 hours post transfection
C
72 hours post transfection

Figure S4

uORF-targeting steric block ASOs do not alter *RNASEH1* mRNA levels at 24, 48, and 72 hours post transfection.

HeLa cells were transfected with 100 nM ASOs or matched chemistry controls and cells harvested at (**A**) 24 hours, (**B**) 48 hours, or (**C**) 72 hours post transfection. *RNASEH1* transcript levels were determined by RT-qPCR and normalised to *RPL10* expression. The value of untreated control samples is indicated by the dotted line (scaled to a value of 1). A gapmer targeting *MALAT1* was included as a positive control for transfection, which is not expected to influence *RNASEH1* expression. Values are mean+SD. Statistical significance was assessed by paired Student's *t*-test between each treatment and its respective control ASO, (no significant changes detected), *n*=3 completely independent experiments.

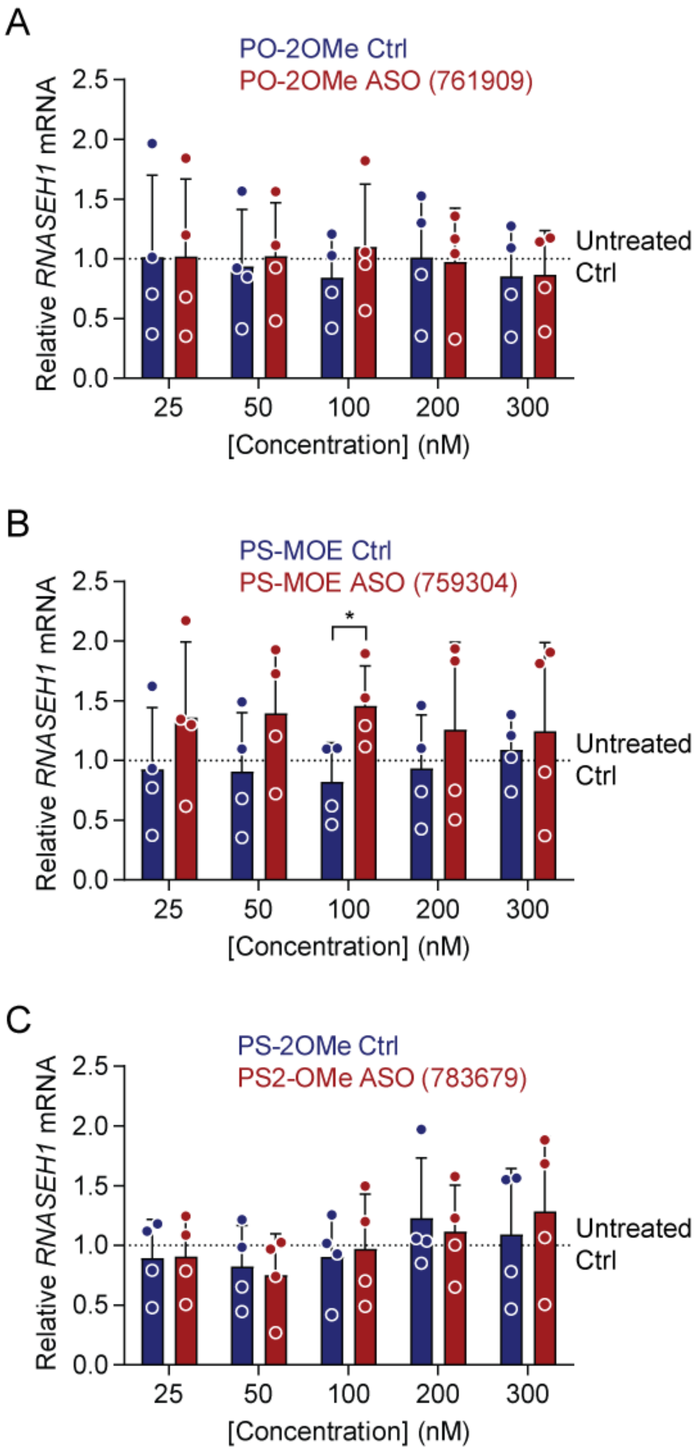
A
PO-2OMe Ctrl
PO-2OMe ASO (761909)
Relative RNASEH1 mRNA
2.5
2.0
1.5
1.0
0.5
0.0
Untreated Ctrl
25
50
100
200
300
[Concentration] (nM)
B
PS-MOE Ctrl
PS-MOE ASO (759304)
Relative RNASEH1 mRNA
*
Untreated Ctrl
[Concentration] (nM)
C
PS-2OMe Ctrl
PS2-OMe ASO (783679)
Relative RNASEH1 mRNA
Untreated Ctrl
[Concentration] (nM)

Figure S5

Expression of *RNASEH1* mRNA levels after treatment with uORF-targeting steric block ASOs at various doses.

HeLa cells were transfected with ASOs at concentrations as indicated and protein harvested after 48 hours for (**A**) PO-2OMe, (**B**) PS-MOE, and (**C**) PS-2OMe nucleic acid chemistries. *RNASEH1* transcript levels were determined by RT-qPCR and normalised to *RPL10* expression. The value of untreated control samples is indicated by the dotted line (scaled to a value of 1). Values are mean+SD. Statistical significance for protein data were assessed by paired Student's *t*-test within each oligonucleotide dose. *P<0.05, n=4 independent experiments

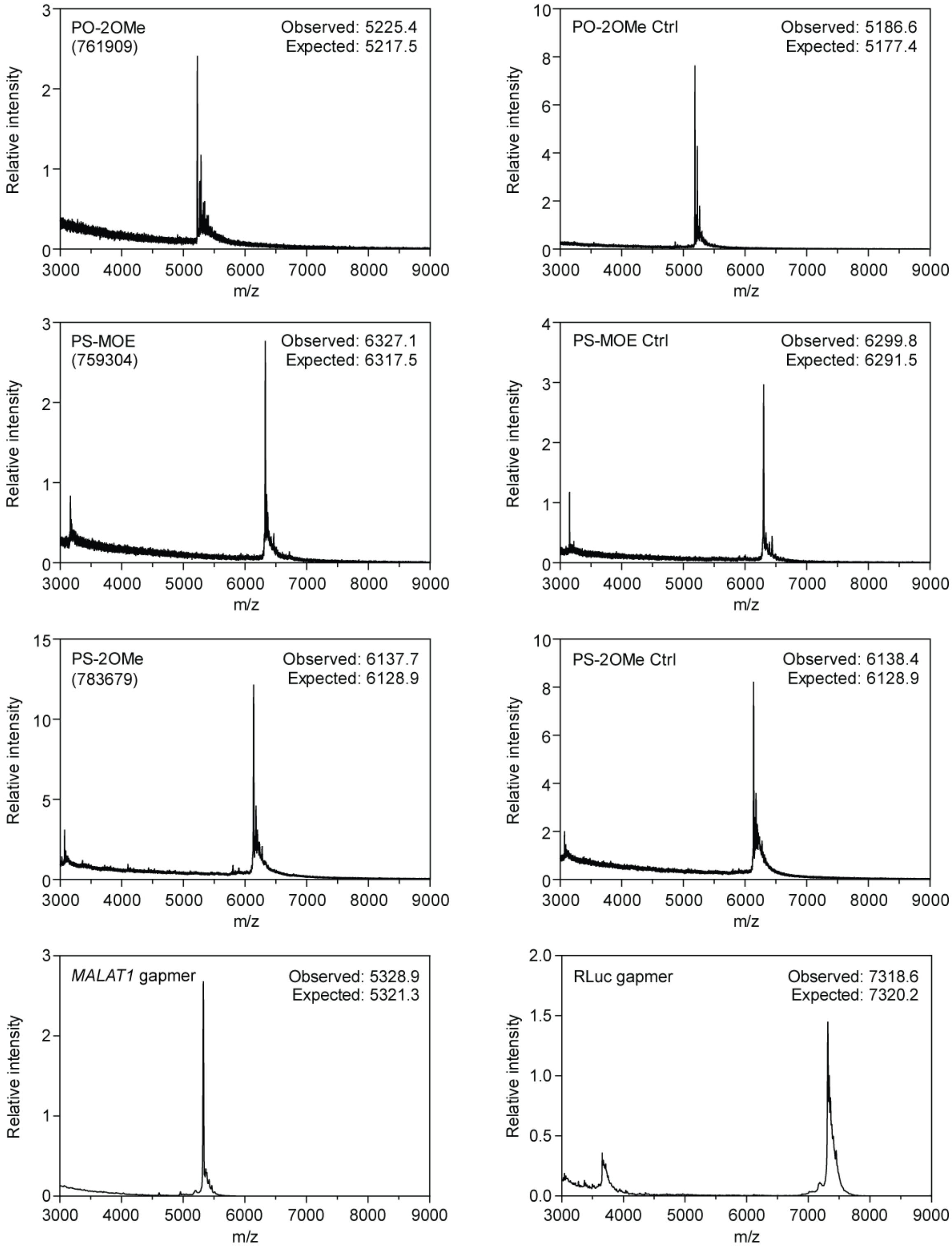
PO-2OMe
(761909)
Observed: 5225.4
Expected: 5217.5
PO-2OMe Ctrl
Observed: 5186.6
Expected: 5177.4
PS-MOE
(759304)
Observed: 6327.1
Expected: 6317.5
PS-MOE Ctrl
Observed: 6299.8
Expected: 6291.5
PS-2OMe
(783679)
Observed: 6137.7
Expected: 6128.9
PS-2OMe Ctrl
Observed: 6138.4
Expected: 6128.9
MALAT1 gapmer
Observed: 5328.9
Expected: 5321.3
RLuc gapmer
Observed: 7318.6
Expected: 7320.2
Relative intensity
m/z

Figure S6

Confirmation of oligonucleotide integrity by MALDI-TOF-MS.

MALDI-TOF-MS spectra for oligonucleotides used in this study. Observed and expected mass values are indicated.

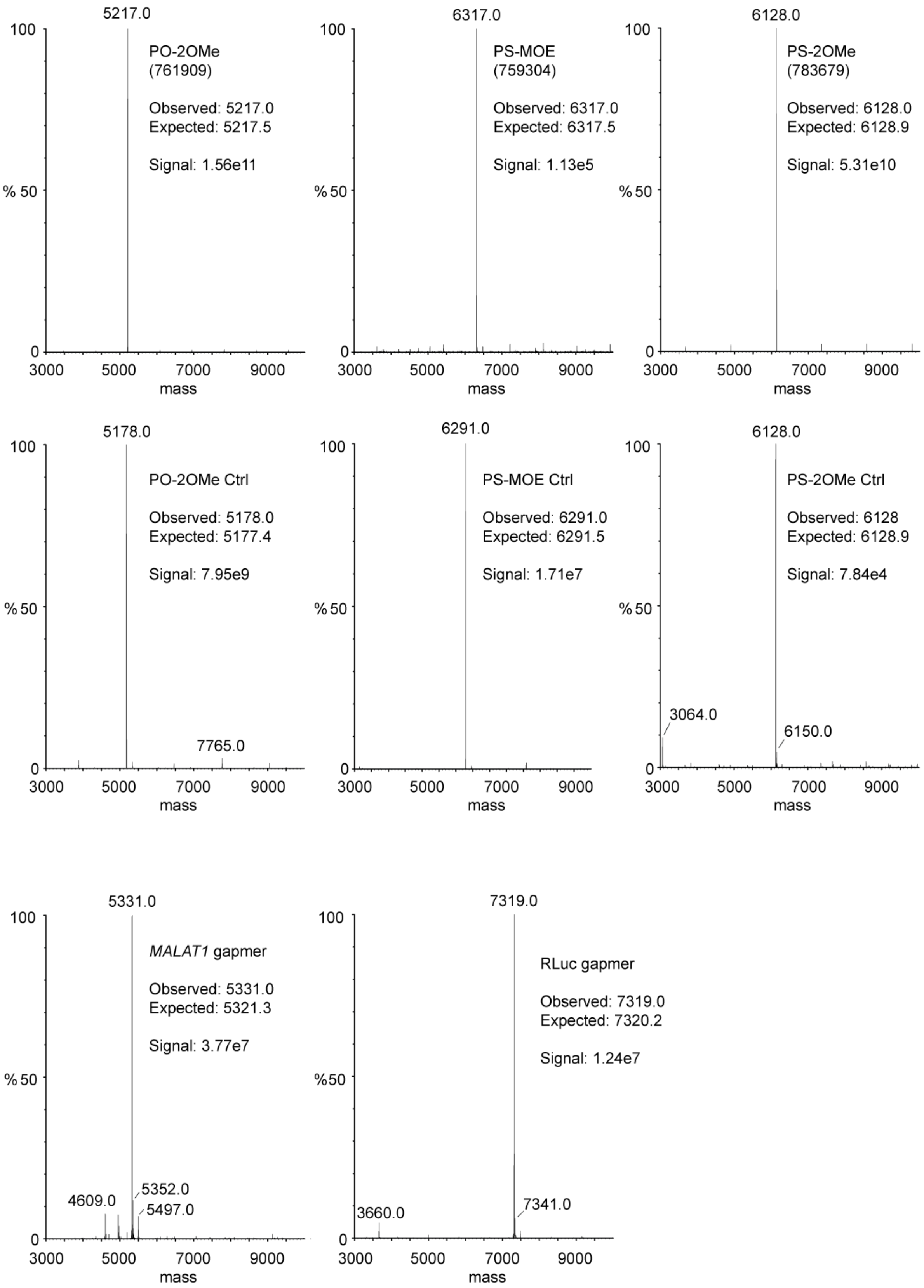
5217.0
PO-2OMe
(761909)
Observed: 5217.0
Expected: 5217.5
Signal: 1.56e11
6317.0
PS-MOE
(759304)
Observed: 6317.0
Expected: 6317.5
Signal: 1.13e5
6128.0
PS-2OMe
(783679)
Observed: 6128.0
Expected: 6128.9
Signal: 5.31e10
5178.0
PO-2OMe Ctrl
Observed: 5178.0
Expected: 5177.4
Signal: 7.95e9
7765.0
6291.0
PS-MOE Ctrl
Observed: 6291.0
Expected: 6291.5
Signal: 1.71e7
6128.0
PS-2OMe Ctrl
Observed: 6128
Expected: 6128.9
Signal: 7.84e4
3064.0
6150.0
5331.0
MALAT1 gapmer
Observed: 5331.0
Expected: 5321.3
Signal: 3.77e7
4609.0
5352.0
5497.0
7319.0
RLuc gapmer
Observed: 7319.0
Expected: 7320.2
Signal: 1.24e7
3660.0
7341.0
100
% 50
0
3000
5000
7000
9000
mass

Figure S7

Confirmation of oligonucleotide integrity by LC-MS.

Mass spectra for oligonucleotides used in this study as analysed by LC-MS. ASO samples were adjusted to 40 µM in water in a total volume of 50 µl prior to separation. Observed and expected mass values are indicated. Electrospray signal intensity is shown for each spectrum.

Table S1

Sequences of oligonucleotides used in this study.

All sequences are written 5′ to 3′. Sequences are provided in IDT notation and as the unmodified sequence only.

**RNASEH1-PO2Me (761909)**	
mCmAmUmUmUmCmGmAmCmUmCmCmCmGmGmC	CAUUUCGACUCCCGGC
**RNASEH1-PO2Me-Control**	
mCmGmCmUmUmCmAmCmUmGmCmCmUmAmCmC	CGCUUCACUGCCUACC
**RNASEH1-PSMOE (759304)**	
/52MOErC/*/i2MOErA/*/i2MOErT/*/i2MOErT/*/i2MOErT/*/i2MOErC/*/i2MOErG/*/i2MOErA/*/i2MOErC/*/i2MOErT/*/i2MOErC/*/i2MOErC/*/i2MOErC/*/i2MOErG/*/i2MOErG/*/32MOErC/	CATTTCGACTCCCGGC
**RNASEH1-PSMOE-Control**	
/52MOErC/*/i2MOErG/*/i2MOErC/*/i2MOErT/*/i2MOErT/*/i2MOErC/*/i2MOErA/*/i2MOErC/*/i2MOErT/*/i2MOErG/*/i2MOErC/*/i2MOErC/*/i2MOErT/*/i2MOErA/*/i2MOErC/*/32MOErC/	CGCTTCACTGCCTACC
**RNASEH1-PS2Me (783679)**	
mC*mA*mU*mU*mU*mC*mG*mA*mC*mU*mC*mC*mC*mG*mG*mC*mC*mC	CAUUUCGACUCCCGGCCC
**RNASEH1-PS2Me-Control**	
mC*mG*mC*mU*mU*mC*mA*mC*mU*mG*mC*mC*mU*mA*mC*mC*mG*mC	CGCUUCACUGCCUACCGC
***MALAT1* gapmer**	
+C*+T*+A*G*T*T*C*A*C*T*G*A*A*+T*+G*+C	CTAGTTCACTGAATGC
**RLuc gapmer**	
/52MOErT/*/i2MOErG/*/i2MOErT/*/i2MOErA/*/i2MOErG/*G*A*G*T*A*G*T*G*A*A*/i2MOErA/*/i2MOErG/*/i2MOErG/*/i2MOErC/*/32MOErC/	TGTAGGAGTAGTGAAAGGCC

Table S2

RT-qPCR assays used in this study.

All sequences are written 5′ to 3′.

Target	Forward	Reverse	Efficiency
RNASEH1	GCAGACAAACCAAAGAGCG	TCCAACCTTGAACCCAGTTAG	1.8240
MALAT1	GCGTAATGGAAAGTAAAGCCC	CAAACACCTCACAAAACCCC	1.8265
RPL10	CCTCTTTCCCTTCGGTGTG	AATCTTGGCATCAGGGACAC	1.8030
**RLuc**	GTAACGCTGCCTCCAGCTAC	CCAAGCGGTGAGGTACTTGT	1.6530
**FLuc**	ACTCTAAGACCGACTACCAGG	GTAGACCCAGAGCTGTTCATG	1.8110
